# Open software platform for automated analysis of paper-based microfluidic devices

**DOI:** 10.1038/s41598-020-67639-6

**Published:** 2020-07-09

**Authors:** Rayleigh W. Parker, Daniel J. Wilson, Charles R. Mace

**Affiliations:** Department of Chemistry, Tufts University, 62 Talbot Avenue, Medford, MA 021551 USA

**Keywords:** Bioanalytical chemistry, Imaging studies, Lab-on-a-chip, Microfluidics, Medical and clinical diagnostics

## Abstract

Development of paper-based microfluidic devices that perform colorimetric measurements requires quantitative image analysis. Because the design geometries of paper-based microfluidic devices are not standardized, conventional methods for performing batch measurements of regularly spaced areas of signal intensity, such as those for well plates, cannot be used to quantify signal from most of these devices. To streamline the device development process, we have developed an open-source program called ColorScan that can automatically recognize and measure signal-containing zones from images of devices, regardless of output zone geometry or spatial arrangement. This program, which measures color intensity with the same accuracy as standard manual approaches, can rapidly process scanned device images, simultaneously measure identified output zones, and effectively manage measurement results to eliminate requirements for time-consuming and user-dependent image processing procedures.

## Introduction

Paper-based microfluidic devices enable measurement capabilities for a number of fields, from clinical diagnostics^1^ to environmental management^2^ and food quality monitoring,^3^ by employing a variety of detection strategies with different signal output formats. These self-contained analytical systems are typically fabricated from paper patterned with hydrophobic barriers, made from materials such as wax,^4^ photoresist,^5^ glue,^6^ or PDMS.^7^ Patterned paper layers can be stacked^8^ or folded^9^ to create three-dimensional fluidic networks, which enable measurement of target analytes by automating complex liquid handling protocols. Depending on the selected signal formation strategy and analysis method, paper-based microfluidic devices can provide qualitative, semi-quantitative, or quantitative results^10.^.

Paper-based platforms that employ electrochemical,^11^ fluorescence,^12^ and chemiluminescence^13^ detection strategies enable quantitative measurements, but generally require secondary equipment, such as a portable potentiostat^14^ or handheld UV source.^15^ To enable measurements without any requirements for specialized external equipment, many developers design devices using colorimetric detection strategies. Qualitative measurements (i.e., on/off sensors) may be interpreted by visual inspection,^8,16^ and readout zones may be compared to printed read guides^1^ or designed to provide distance-based outputs^17–20^ to enable semi-quantitative measurements. Image analysis is used to characterize assay performance^21^ during the device development process, but can also be performed at the point-of-use by smartphone applications^22^ to provide quantitative measurements while reducing user training requirements.^23^ These applications may operate using algorithms that are specific to the geometry of the device being analyzed or from unpublished code that is not available for modification,^24^ requiring developers of paper-based devices to develop their own software tools or rely on manual image analysis protocols.

As a critical component of paper-based assay development, especially for qualitative and semi-quantitative devices, image analysis facilitates investigation of device design criteria that determine how a user may interpret developed signal. During the prototyping process, device readout zones are typically imaged using a flatbed scanner or camera, and the acquired images are analyzed to inform device fabrication and assay conditions to provide sufficient analytical performance of the device. Images of device output zones are often measured using free and open-source tools (e.g., ImageJ^25^, Fiji^26^) that support user-developed plugins^27,28^ for application-specific analyses. Although numerous plugins exist, available options do not facilitate streamlined analysis of paper-based output zones of different colors and geometries. While manual approaches for image analysis of colorimetric signals have broad utility for general measurement needs across many fields, application of these tools for analysis of paper-based devices is labor-intensive, user dependent, and time consuming. Our program effectively packages the capabilities of existing general color analysis techniques and applies them towards solving the specific challenges facing the interpretation of paper-based assays (e.g., non-standardized zone numbers and geometries).

For example, to analyze a paper-based output zone in ImageJ, a user must first define a region of interest (ROI) for analysis (e.g., using the “Oval” tool for a circular zone). This region is typically defined on a magnified view of a high-resolution (e.g., 600–800 dpi)^8,29^ image. At high magnification, it can be difficult for a user to differentiate between the signal-containing area and surrounding material (e.g., hydrophobic wax barrier). To avoid introducing bias in measured signal intensities, this region must also be centered on the output zone so that the selected area does not contain any undesired surrounding material and adequately captures any non-uniform distribution of signal. When the ROI has been placed in the desired location, the output zone may be analyzed by a selected method (e.g., “RGB Measure”)^30^. After a single measurement is complete, the ROI can be moved to or recreated on the next output zone so that the measurement process can be repeated. The area and placement of this region must be consistent throughout the analysis process so that measurements are consistent across output zones. Measured values can then be copied or exported for further statistical analysis. The reproducibility of these results may vary by software user, as size and placement of the ROI are both manually defined for individual measurements.

Because the device development process typically requires analysis of replicate results across many conditions, potentially necessitating hundreds of devices, image analysis and data processing can be substantially labor-intensive and time-consuming for device developers. For complex devices, these requirements can inhibit broad screening of fabrication or use conditions (e.g., channel geometry, reagent storage, sample volume). Automated image processing can improve the time requirements and precision of measured results for colorimetric paper-based assays, but existing ImageJ plugins and available tools are not compatible with device-specific design geometries^31^ and spatial arrangements of color localization in most paper-based devices.

Existing ImageJ plugins, such as “ReadPlate”^32^, enable automated analysis of images of well plates with standard configurations (e.g., 96-well plates). This plugin streamlines analysis by allowing the user to define a grid of circular regions of interest that is superimposed on the well plate image. The grid is created by defining the number of rows and columns in the well plate, the pixel coordinates of bounding wells (e.g., wells A1 and H12), and the diameter of each analysis spot. Because paper-based devices are designed in custom, non-linear geometries^33^ according to their intended performance and function, their output zones typically do not follow the spatial arrangement of commercial liquid handling tools. Other ImageJ plugins, including “Template Matching”^34^ and “Template Matching and Slice Alignment”^35^, can perform automated recognition of desired image features based on a user-generated reference template or selection. These tools are designed to recognize the extent of agreement between a reference template and a larger image and may not be sufficient for recognizing multiple shapes or colors within a single image.

Since the development of early paper-based devices, cellular phones have been used to enable quantitative analysis of colorimetric signal^21^. As smartphone technologies and quantitative measurement accessories^36,37^ have advanced over time, many groups have written custom applications to quantify signal from paper-based devices at the point-of-care^22,38–42^. Smartphone image analysis applications are typically tailored to the geometries of individual paper-based assays^43^ and cannot be used to measure output zones that differ from those of the original device. In many cases, the positions of test zones are detected using registration marks patterned within the paper device^22,44^. These recognition algorithms do not independently identify the positions of signal formation and instead analyze known areas of signal formation. Additionally, the source code for these applications is not always published with scientific manuscripts^24^, and the resulting lack of modifiable open-source options requires developers of paper-based devices to either (i) create their own analysis software or (ii) rely on existing inefficient options throughout the assay development process.

To address this shortcoming, we have developed a free, open-source software called ColorScan that enables streamlined, automated analysis of paper-based microfluidic devices. This Python-based program automatically identifies and measures signal-containing zones of any geometry or color from images of paper-based devices. Our tool provides a variety of quantitative measurement options based on user-specified criteria and effectively manages data, even providing cropped images of output zones paired with measured results to facilitate figure creation. To verify the performance of this software, we compared the consistency and time requirements of our tool to manual measurements completed using ImageJ. Our software, which has the potential to simplify the time and labor-intensive process of quantitative image analysis for paper-based devices, is freely available^31,45,46^, as an easy-to-use Python program to facilitate widespread use and further improvement by other developers of colorimetric sensors.

## Experimental design

### Identification of desired software features

We designed ColorScan to automate the workflow of our image analysis process, which consists of three main steps: (i) selection of a region of interest, (ii) color intensity measurement within the selected region, and (iii) management of measured results. When a paper-based device is imaged to facilitate analysis, arbitrary placement or rotation of the device on the scanner bed or within the camera’s field of view can lead to variability of output zone position across replicate devices. Manual selection of analysis regions can be performed regardless of zone position, but is time consuming, while patterned registration marks for automated analysis programs place design constraints on device developers. We designed ColorScan to automatically recognize colored regions of an image based on hue (i.e., the color or shade of the output zone), saturation (i.e., amount of gray), and value (i.e., the brightness of the output zone). This recognition step is not dependent on the spatial location of colored pixels within the image file, enabling automated analysis of devices imaged in any orientation.

Manual analysis protocols, in addition to being labor-intensive and user-dependent, usually only allow an image to be measured within a single color space. Device images are typically acquired in the RBG color space, requiring conversion to determine if another color space (e.g., HSV, CIELAB)^47,48^ is more sensitive to the signal formed by a particular sensor or more intuitive for interpretation by visual inspection^49^. Standard image analysis approaches also require measurement results to be manually tracked and compiled into spreadsheets for processing. We developed ColorScan to not just automate analysis in multiple color spaces, but also effectively manage results by organizing them in a common location (i.e., a .csv file) for direct comparison during the assay development process. To streamline comparison of measurement results to their respective output zones, as well as process device images for presentation or publication, we configured ColorScan to crop and save an image of each measured output zone. The file name of each image is labeled so that it may be paired with its numerical measurement results.

### Computational analysis approach

To make our software broadly accessible and readily modifiable, we chose to develop it using Python^50^, a programming language that is used in a wide range of fields, has a large assortment of libraries, and is compatible with a number of third-party modules. We used OpenCV^51^, an extensive open-source computer vision library, in conjunction with NumPy^52^, an array manipulation and numerical operation library, to perform image processing tasks. We also developed a graphical user interface, using the Python TkInter library^53^, to ensure that ColorScan would be accessible to a broad user base without requiring device developers to be experienced in coding. The source script for our Python-based program is available for download at our group’s GitHub page^54^ and we have included a detailed *User Guide* document, including instructions for downloading and using Python, as part of the Supplementary Information.

**Figure 1:**
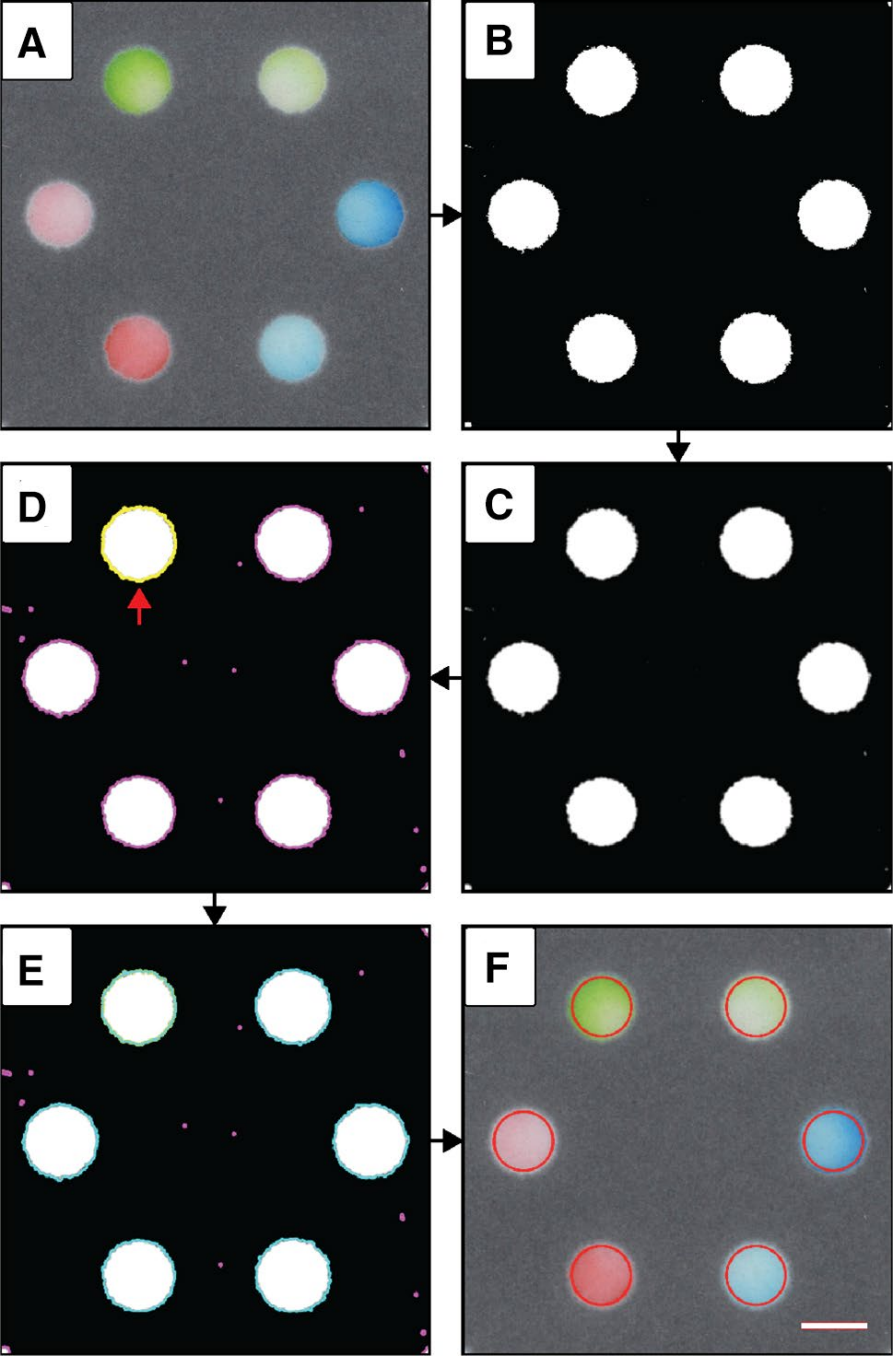
ColorScan image analysis protocol. (**A**) Scanned image of paper-based microfluidic device output zones filled with dyes. (**B**) A binarized image generated using value and saturation masking thresholds identifies areas of the image that contain colorimetric signal. (**C**) The masked image is blurred to smooth zone boundaries before contour detection. (**D**) A contour identification step highlights the borders of all white areas within the image (magenta). A reference contour (yellow) can be selected for identification of similar contours. (**E**) Identified contours that are sufficiently similar in size and shape to the reference contour are highlighted in light blue. (**F**) An analysis region defined within the reference contour is applied to all similar contours to support batch measurement of identical analysis regions. Scale bar is 4 mm.

The first step in our image analysis protocol (Fig. 1A) is identification of the colorimetric signal contained by the output zones of a paper-based device. We designed ColorScan to automatically recognize the color of output zones against the contrasting color of the patterned hydrophobic barrier, which is black wax in our devices. The software masks the area around the zones by temporarily converting the image to the HSV (i.e., Hue, Saturation, Value) color-space, and excluding pixels below minimum saturation and value thresholds defined by the user. This masking step produces a binarized image (Fig. 1B), in which color-containing pixels within the device output zones are identified for further refinement.

To reduce the jaggedness of the masked output zone edges, ColorScan performs a box blur (Fig. 1C) based on a user-defined kernel size. Next, the software runs OpenCV’s contour-identification algorithm, which is based on work by Suzuki and Abe^55^, on the binarized image to define the boundaries of the areas where color intensity measurements may be performed (Fig. 1D). In order to cut down on computation time, we set an arbitrary cutoff for contours containing fewer than five pixels in area. This threshold effectively determines the minimum feature size that ColorScan can detect. The open-source nature of the code, however, allows the user to modify this threshold to suit their needs. The maximum feature size is, in principle, the size of the image itself. At this point, the user selects a reference contour to facilitate identification of similar objects for analysis. ColorScan compares the sizes (i.e., pixel area contained within the border) and shapes, defined by the Hu image invariants^56^, of the reference contour and all of the identified contours based on thresholds set by the user in the graphical user interface. In this comparison, the OpenCV shape-matching function computes the sum of absolute differences between each of the seven Hu invariants for the two contours under comparison (i.e., the reference contour and another contour), and produces a score indicating how similar the two contours are. Contours with scores within the user-defined thresholds are selected for batch analysis (Fig. 1E).

For device designs that use a colored hydrophobic barrier feature to provide contrast for visual interpretation of assay results^8,57^, the software may recognize these features as part of the output zone. To ensure that inclusion of barrier edge pixels does not bias measurement results, we developed a zone refinement tool that allows the user to geometrically constrain the reference contour to exclude undesired image features. This constraint needs only be manually performed on the reference contour and is applied to all similar contours before analysis. This feature also allows the user to select regular polygonal geometries, in addition to common circular output zones. Performing this step provides direct control of the exact size and position of each analysis region in a batch measurement (Fig. 1F).

Once the user is satisfied that all of the output zones—and only the output zones—have been selected, the software will measure all of the pixels within each contour. Results can be presented as average color intensities in up to three color spaces, including RGB, HSV, and CIELAB, and also as histograms of RGB intensities, depending on user-defined preferences. Measurements are exported to a .csv file and may be compared to saved images of output zones, which are automatically cropped from the full image based on the bounding rectangle of each output zone contour and saved with their associated index identifier to facilitate data curation and presentation.

### Design of software and graphical user interface

**Figure 2:**
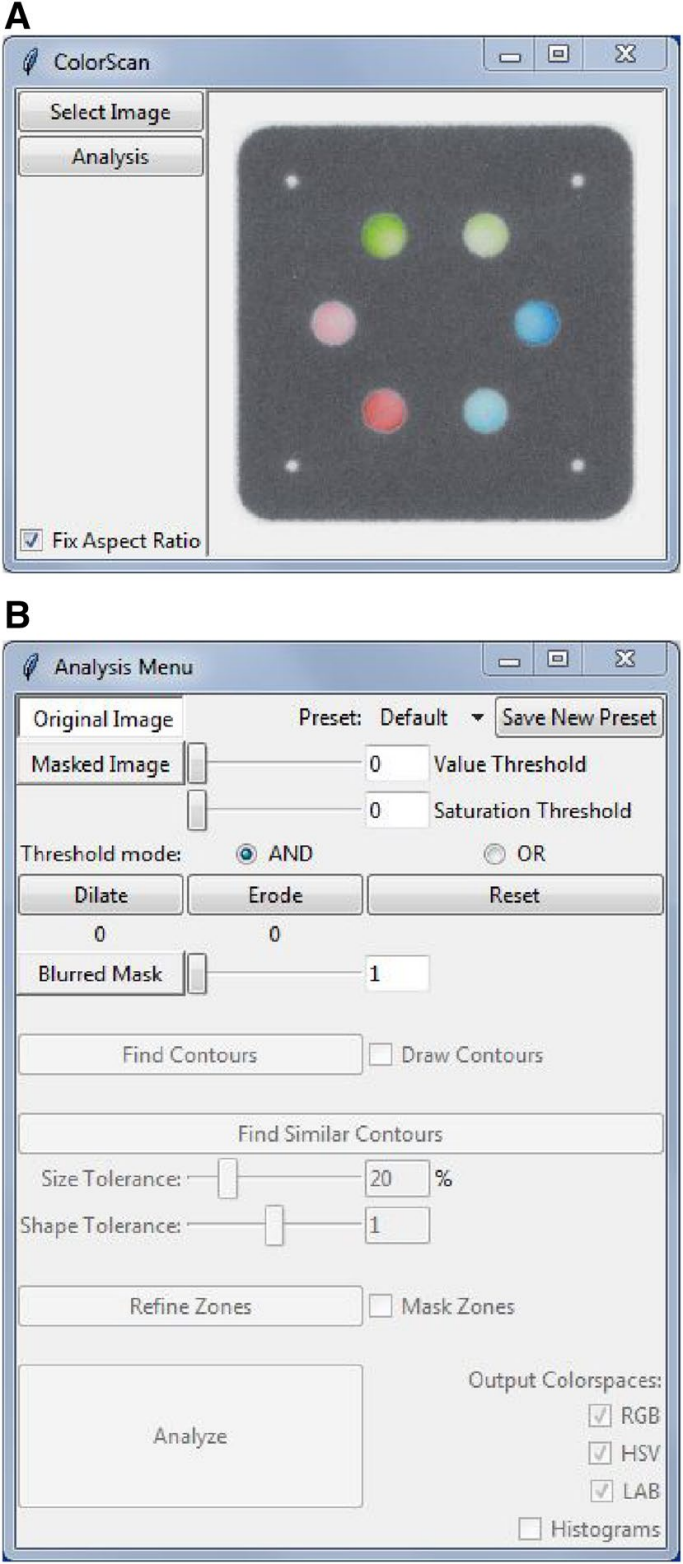
ColorScan graphical user interface. (**A**) The main ColorScan window displays the image being analyzed. (**B**) The Analysis Menu window contains all of the analysis control options. Additional features of this window become usable as the measurement process is completed.

We designed ColorScan to have a simple graphical user interface that would streamline the image analysis process and improve consistency across users. The first step in using ColorScan is selecting an image. Selecting an image using the “Select Image” button displays it in the main window of the software (Fig. 2A). The program supports most image file types, the full list of which can be found in the OpenCV documentation^58^. The image in the main ColorScan window updates as options in the Analysis Menu window (Fig. 2B) are adjusted. This window is opened with the “Analysis” button after image selection, and its features are spatially arranged, from top to bottom, in the sequence that they should be used to complete image analysis. At the beginning of the analysis process, only the masking controls are available in the Analysis Menu window. Additional features become available as each step in the analysis sequence is completed.

First, the value slider is used to mask the area surrounding the output zones and highlights pixels above a selected brightness threshold. Next, the saturation slider is used to refine the masked areas by filtering unsaturated pixels (i.e., those close to grayscale) to show only pixels containing color. After identification of the output zones, the blur options can be used to smooth the mask edges and reduce the granularity of the areas to be analyzed. The “Dilate” and “Erode” buttons expand and shrink the masked and blurred areas, respectively, allowing for further refinement before selection of the output zone contours. Masking and blurring parameters can be saved into a named preset, which can be loaded during subsequent analyses to facilitate rapid, consistent analysis of assay replicates.

Once masking parameters include all of the desired output zones, the user can find the contours of those zones using the “Find Contours” button. This will select all color-containing regions in the image, from which the user may select a reference contour by clicking on it. The “Find Similar Contours” button selects all regions that are similar in size and shape to the reference contour for analysis. The Size Tolerance and Shape Tolerance values should be adjusted so that only the device output zones are highlighted. After these zones are selected for analysis, clicking the Refine Zones button will open a separate window that enables geometric restriction of the analysis area within each output zone.

This tool is useful for cases where undesired pixels from the hydrophobic barrier edge (e.g., black wax) or a patterned contrast feature (e.g., colored rings) are selected by the masking and contour finding steps but should not be included in the area that is being measured. To ensure that only signal from the paper-based assay is measured, the tools in the Refine Zone window allow the user to constrain the position, shape, and area of the analysis region within the reference contour. This analysis geometry is applied to all identified similar contours to provide consistent measurement area and shape across all analysis regions. It also allows for better handling of potential problem-cases where there is insufficient contrast between the color-containing regions of interest (i.e., the test zones) and the background color from the device (e.g., colored wax barriers or the paper itself), which may occur when the assay produces colors that are either very dark (low Value) or very pale (low Saturation). By refining the zone area, the user may cut out regions potentially erroneously included in the analysis region. This process can be optimized for a specific device geometry and then saved as a preset to facilitate objective and reproducible measurements across multiple images. These techniques, in combination with judicious device design features (e.g., colored rings printed around output zones to improve contrast) make consistent contour identification possible.

**Figure 3:**
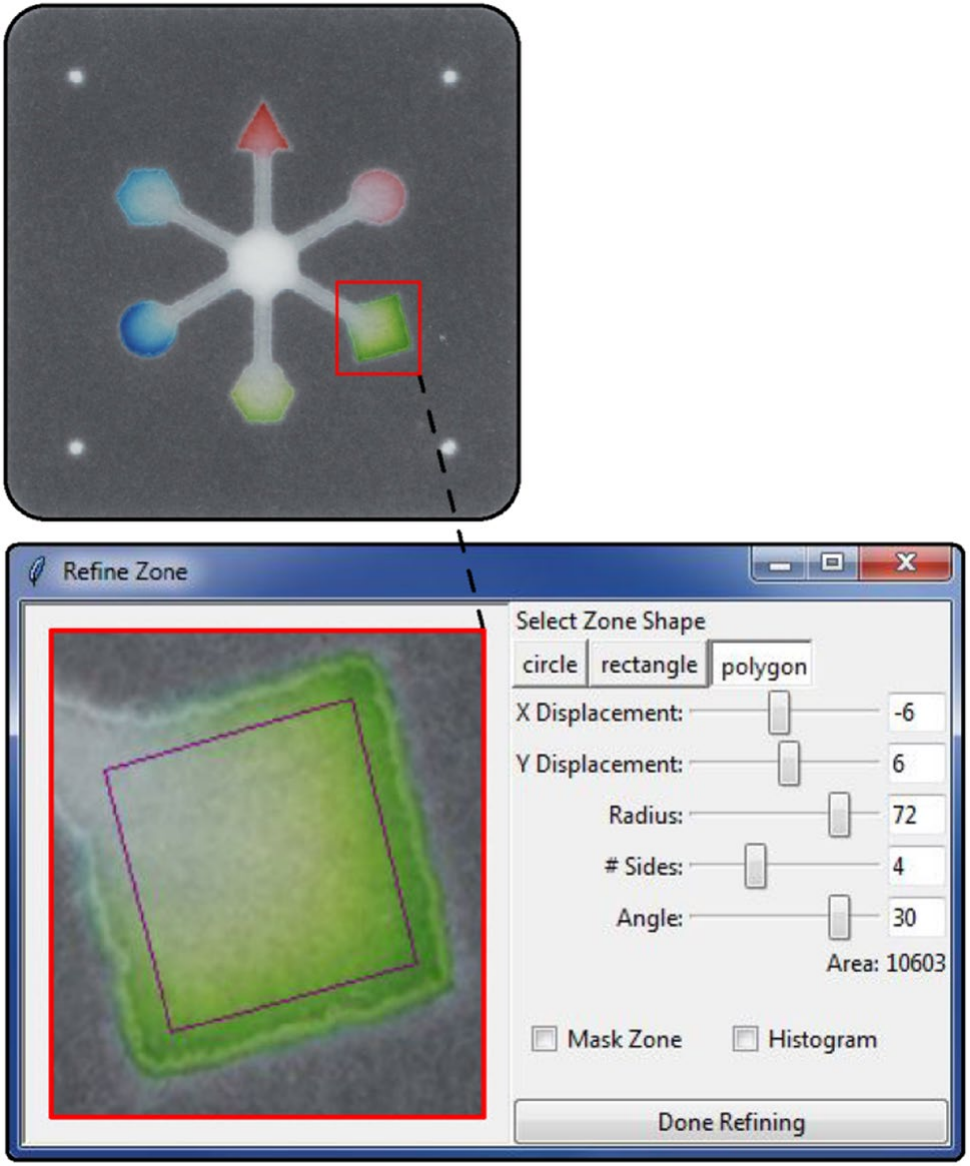
ColorScan zone refinement tool. Detection zones at the ends of paper channels, like the ones shown in the above paper-based microfluidic device, can be precisely selected for measurement using the options in the Refine Zone window. Identical zone selections are propagated to the same relative positions in the contours the user and program have identified as being the same size and shape of the reference contour. This allows for reproducible, precise analyses of replicate assays on multiplexed devices.

Importantly, the positional controls in the Refine Zone window (X Displacement, Y Displacement, Angle) can be used to define the analysis region at the end of a paper channel (Fig. 3). Many device designs^21,22,57^ contain detection zones at the ends of paper channels used for fluid distribution. While the white or grey color of these channels can be difficult to distinguish from colorimetric signal during masking steps in ColorScan, our zone refinement approach allows these features to be analyzed in a controlled, reproducible manner. After zone refinement, pressing the “Analyze” button completes automated measurement of all analysis regions selected by the user-defined criteria and exports results and zone images to the same directory as the original image.

## Results and Discussion

### Color intensity measurements

**Figure 4:**
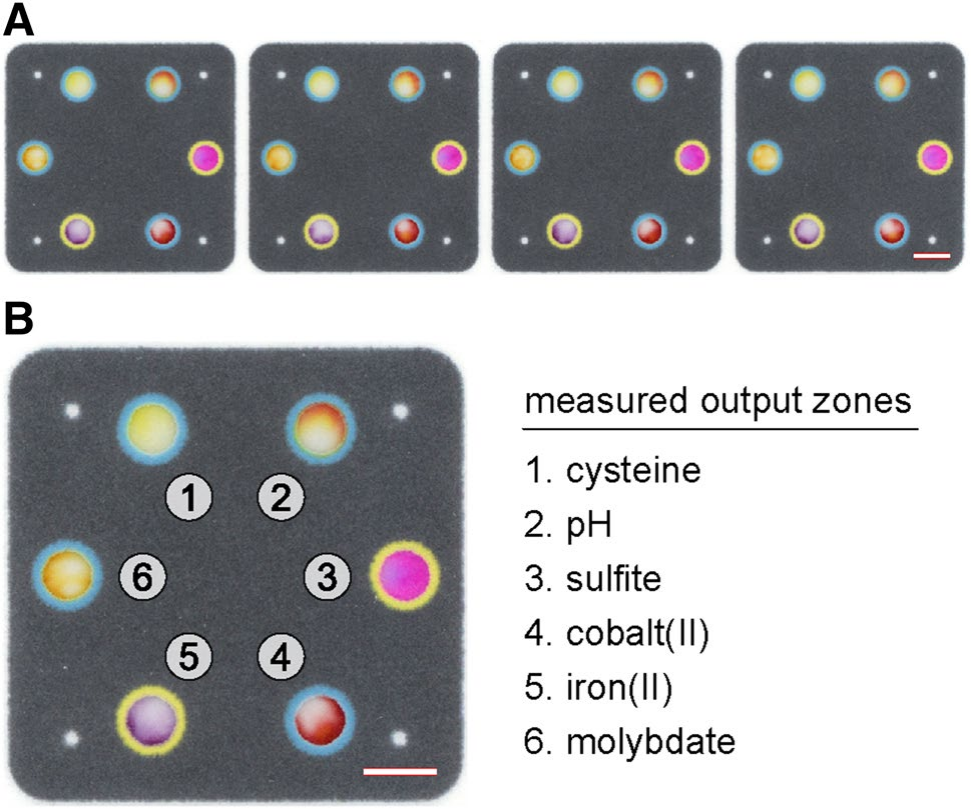
Paper-based microfluidic devices used to complete ColorScan performance evaluation. (**A**) A scanned image of a strip of four replicate devices was measured in the RGB color space using ImageJ and ColorScan. (**B**) Each device contained output zones that provided six different colorimetric signals. These zones were bordered by wax-printed contrast rings, a common feature of paper-based devices that are designed to be interpreted by visual inspection. Scale bars in both (**A**) and (**B**) are 6 mm.

We compared the user experience and measurement results provided by ColorScan to those of our standard image analysis approach, completed using ImageJ, to evaluate the performance of our custom software. To complete this comparison, we analyzed four replicate paper-based microfluidic devices (Fig. 4A) containing six circular output zones each, using both ImageJ and ColorScan. These four-layered devices, described in the *Materials and Methods* document as part of the Supplementary Information, contained internally stored and spatially separated reactants for six colorimetric reactions. When these devices were run with water, stored analytes and their reagents were rehydrated and delivered to output zones where colorimetric signal developed to indicate the presence of (i) cysteine, (ii) a neutral pH, (iii) sulfite, (iv) cobalt(II), (v) iron(II), or (vi) molybdate. Each of these zones was surrounded by a wax-printed contrast ring (Fig. 4B), which could be used to support signal interpretation by visual inspection. These devices were not designed to perform measurements for these analytes at the point-of-care, but the homogenous (e.g., sulfite zone) and heterogeneous (e.g., cobalt(II) zone) distributions of signal intensity in their output zones are demonstrative of signal formation patterns found in practical paper-based microfluidic devices. All devices were imaged using an Epson V600 Photo flatbed scanner at a resolution of 800 dpi. Scanning at lower resolutions is not expected to appreciably bias measurements acquired from a test zone—data from neighboring pixels within a zone that can be resolved in a high-resolution image are effectively accounted for through averaging in a low-resolution image. Practically, we suggest setting 300 dpi as a lower limit for images and scans.

**Figure 5:**
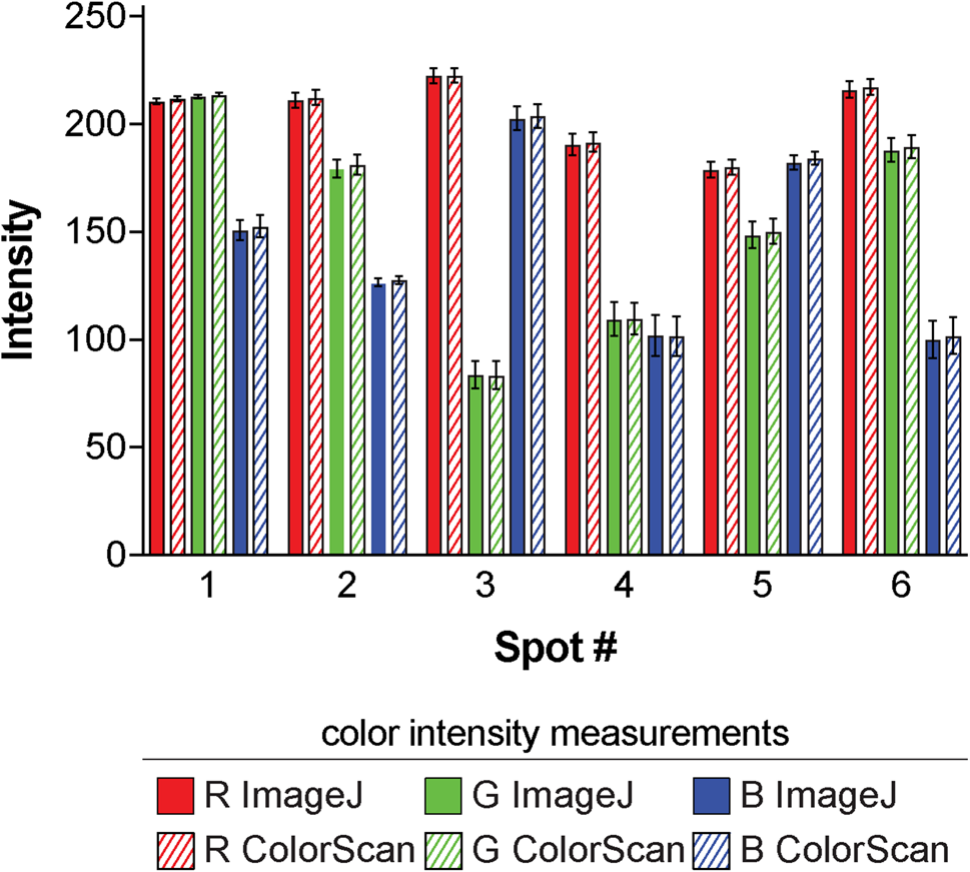
ColorScan performance evaluation. Solid and diagonally striped bars represent ImageJ and ColorScan measurement results, respectively, performed in each channel of the RGB color space. Spot numbers correspond to the diagram shown in Fig. 4B. Error bars represent standard deviation of four replicate measurements of each numbered output zone.

To complete our performance evaluation, we began by manually measuring the pixel intensity of each output zone in ImageJ using the “RGB Measure” tool^30^. We created a circular region of interest on the scanned device image and manually measured the output zones in the numerical order shown in Fig. 4B, then compiled measurement results in a Microsoft Excel spreadsheet. To acquire images of each output zone, as ColorScan does automatically, we manually cropped selected features from the scanned device image using Adobe Photoshop. In total, our manual measurement and image processing steps took approximately 24 min. ColorScan analysis of the same image took only 2 min and automatically provided an organized spreadsheet of results and cropped images of output zones. This is a reasonable analysis time for a trained ColorScan user, and we expect the time investment required for users to familiarize themselves with the software to be minimal. Unlike manual analysis performed using ImageJ, the time required to perform automated measurements in ColorScan does not significantly depend on the number of output zones being measured, offering substantial time savings over conventional methods. Measurement results obtained in the RGB color space using each software are shown in Fig. 5. Further details of our performance evaluation, including additional comparisons for the HSV and CIELAB color spaces, are available in *Materials and Methods* document of the Supplementary Information.

For each set of output zones measured in the RGB color space, mean pixel intensities acquired using ColorScan were consistent with values obtained using ImageJ. On average, ColorScan and ImageJ values were 0.7% different, with a maximum difference of 1.8% for the blue channel intensity of the purple-colored signal in output spot 6 of each device (Table 1). These differences may be related to minor inconsistencies in analysis region position or size in each approach, or the computational methods used to average pixel intensity in each program. Additionally, the variance of mean pixel intensity values for replicate output zones is comparable for measurements performed using ColorScan and ImageJ (Table S3). These results indicate that ColorScan, in addition to providing a user-friendly approach for streamlined image analysis, performs pixel intensity measurements just as well as standard image analysis programs.

**Table 1:** Mean RGB pixel intensities measured using ImageJ and ColorScan. Percent difference calculations used to compare measurement performance of the two programs indicate that ColorScan provides accurate pixel intensity values that are consistent with values measured by ImageJ.

Spot #	1	2	3	4	5	6
	Mean pixel intensity					
**ImageJ**						
Red	211	211	223	191	179	216
Green	213	180	84	110	149	188
Blue	151	127	203	102	182	100
**ColorScan**						
Red	212	212	223	192	180	218
Green	214	181	84	110	150	190
Blue	153	128	204	102	184	102
**% Difference**						
Red	0.6	0.6	0.1	0.6	0.8	0.6
Green	0.5	1.0	0.2	0.2	1.1	0.8
Blue	1.2	1.0	0.5	0.3	1.1	1.8

## Conclusions

We have developed an open-source computer program designed specifically to facilitate the automated analysis of colorimetric signals in paper-based microfluidic devices. This program, ColorScan, independently identifies locations of signal formation in images of devices to enable simultaneous measurement of replicate output zones arrayed in any geometry. We intended this approach to address outstanding challenges facing the development of paper-based assays: (i) Because assays conducted in paper devices are not restricted to any set zone size, shape, or spatial orientation, the time-intensive methods used for manual data analysis can create an arbitrary obstacle to conducting large numbers of replicates or comprehensively evaluating device design criteria. (ii) The use of presets enables reproducible analyses that are scalable. ColorScan operates identically if an image contains few or numerous zones, if an image contains numerous devices, or if data sets span numerous images. ColorScan requires minimal user intervention and offers a substantial time savings over manual image analysis methods (e.g., via ImageJ) and provides accurate and reproducible pixel intensity measurements in multiple color spaces (RGB, HSV, and CIELAB). Moreover, ColorScan effectively manages measurement results by (i) tagging each identified test zone with a unique identifier; (ii) exporting measurements to a .csv file; (iii) creating histograms, as both .csv tables and .png plot images, of values within each zone, (iv) compiling averages and standard deviations for each zone and color space, (v) generating cropped and centered images of each logged zone to facilitate the design of publication-quality figures. By automating user-dependent aspects of the image analysis process, this tool improves the consistency and speed of color intensity measurements to support evaluation of assay conditions and design criteria during the development of paper-based microfluidics. Finally, as an open source tool, ColorScan can facilitate transparency of data analysis techniques.

## Supplementary information

Supplementary file1 (PDF 3532 kb)

Supplementary file2 (PDF 4936 kb)

## References

[1] Pollock NR (2012). A paper-based multiplexed transaminase test for low-cost, point-of-care liver function testing. Sci. Transl. Med..

[2] Hofstetter JC (2018). Quantitative colorimetric paper analytical devices based on radial distance measurements for aqueous metal determination. Analyst.

[3] Jokerst JC (2012). Development of a paper-based analytical device for colorimetric detection of select foodborne pathogens. Anal. Chem..

[4] Carrilho E, Martinez AW, Whitesides GM (2009). Understanding wax printing: a simple micropatterning process for paper-based microfluidics. Anal. Chem..

[5] Martinez AW, Phillips ST, Butte MJ, Whitesides GM (2007). Patterned paper as a platform for inexpensive, low-volume, portable bioassays. Angew. Chem. Int. Ed..

[6] Cardoso TMG (2017). Versatile fabrication of paper-based microfluidic devices with high chemical resistance using scholar glue and magnetic masks. Anal. Chim. Acta.

[7] Bruzewicz DA, Reches M, Whitesides GM (2008). Low-cost printing of poly(dimethylsiloxane) barriers to define microchannels in paper. Anal. Chem..

[8] Schonhorn JE (2014). A device architecture for three-dimensional, patterned paper immunoassays. Lab Chip.

[9] Liu H, Crooks RM (2011). Three-dimensional paper microfluidic devices assembled using the principles of origami. J. Am. Chem. Soc..

[10] Fernandes SC, Walz JA, Wilson DJ, Brooks JC, Mace CR (2017). Beyond wicking: expanding the role of patterned paper as the foundation for an analytical platform. Anal. Chem..

[11] Nie Z (2010). Electrochemical sensing in paper-based microfluidic devices. Lab Chip.

[12] Carrilho E, Phillips ST, Vella SJ, Martinez AW, Whitesides GM (2009). Paper microzone plates. Anal. Chem..

[13] Ge L, Wang S, Song X, Ge S, Yu J (2012). 3D Origami-based multifunction-integrated immunodevice: low-cost and multiplexed sandwich chemiluminescence immunoassay on microfluidic paper-based analytical device. Lab Chip.

[14] Delaney JL, Doeven EH, Harsant AJ, Hogan CF (2013). Use of a mobile phone for potentiostatic control with low cost paper-based microfluidic sensors. Anal. Chim. Acta.

[15] Connelly JT, Rolland JP, Whitesides GM (2015). “Paper machine” for molecular diagnostics. Anal. Chem..

[16] Kim S, Sikes HD (2019). Liposome-enhanced polymerization-based signal amplification for highly sensitive naked-eye biodetection in paper-based sensors. ACS Appl. Mater. Interfaces.

[17] Pratiwi R (2017). A selective distance-based paper analytical device for copper(II) determination using a porphyrin derivative. Talanta.

[18] Yamada K, Citterio D, Henry CS (2018). “Dip-and-read” paper-based analytical devices using distance-based detection with color screening. Lab Chip.

[19] Gerold CT, Bakker E, Henry CS (2018). Selective distance-based K^+^ quantification on paper-based microfluidics. Anal. Chem..

[20] Berry SB, Fernandes SC, Rajaratnam A, DeChiara NS, Mace CR (2016). Measurement of the hematocrit using paper-based microfluidic devices. Lab Chip.

[21] Martinez AW (2008). Simple telemedicine for developing regions: camera phones and paper-based microfluidic devices for real-time, off-site diagnosis. Anal. Chem..

[22] Lopez-Ruiz N (2014). Smartphone-based simultaneous pH and nitrite colorimetric determination for paper microfluidic devices. Anal. Chem..

[23] Phillips EA, Moehling TJ, Bhadra S, Ellington AD, Linnes JC (2018). Strand displacement probes combined with isothermal nucleic acid amplification for instrument-free detection from complex samples. Anal. Chem..

[24] Dryden MDM, Fobel R, Fobel C, Wheeler AR (2017). Upon the shoulders of giants: open source hardware and software in analytical chemistry. Anal. Chem..

[25] ImageJ. https://imagej.nih.gov/ij/ (Accessed August 13, 2019).

[26] Fiji: ImageJ, with "Batteries Included". https://fiji.sc/ (Accessed August 13, 2019).

[27] Guzmán C, Bagga M, Kaur A, Westermarck J, Abankwa D (2014). ColonyArea: an ImageJ plugin to automatically quantify colony formation in clonogenic assays. PLoS ONE.

[28] Della Mea V, Baroni GL, Pilutti D, Di Loreto C (2017). SlideJ: An ImageJ plugin for automated processing of whole slide images. PLoS ONE.

[29] Martinez AW, Phillips ST, Whitesides GM (2008). Three-dimensional microfluidic devices fabricated in layered paper and tape. Proc. Natl. Acad. Sci. USA.

[30] Rasband, W. RGB Measure. https://imagej.nih.gov/ij/plugins/rgb-measure.html (2004).

[31] DeChiara NS, Wilson DJ, Mace CR (2017). An open software platform for the automated design of paper-based microfluidic devices. Sci. Rep..

[32] Delfino, J. M. ReadPlate. https://imagej.nih.gov/ij/plugins/readplate/index.html (2015).

[33] Wilson DJ, Mace CR (2017). Reconfigurable pipet for customized, cost-effective liquid handling. Anal. Chem..

[34] O’Dell, W. Template matching. https://imagej.nih.gov/ij/plugins/template-matching.html (2005).

[35] Tseng, Q. Template matching and slice alignment—ImageJ plugins. https://sites.google.com/site/qingzongtseng/template-matching-ij-plugin#downloads (Accessed August 14, 2019).

[36] Zhu H, Yaglidere O, Su T-W, Tseng D, Ozcan A (2011). Cost-effective and compact wide-field fluorescent imaging on a cell-phone. Lab Chip..

[37] Laksanasopin T (2015). A smartphone dongle for diagnosis of infectious diseases at the point of care. Sci. Transl. Med..

[38] Guan L (2014). Barcode-like paper sensor for smartphone diagnostics: an application of blood typing. Anal. Chem..

[39] Delaney JL, Hogan CF, Tian J, Shen W (2011). Electrogenerated chemiluminescence detection in paper-based microfluidic sensors. Anal. Chem..

[40] Jalal UM, Jin GJ, Shim JS (2017). Paper–plastic hybrid microfluidic device for smartphone based colorimetric analysis of urine. Anal. Chem..

[41] Güder F (2016). Paper-Based Electrical Respiration Sensor. Angew. Chem. Int. Ed..

[42] Choi JR (2016). An integrated paper-based sample-to-answer biosensor for nucleic acid testing at the point of care. Lab Chip.

[43] Yetisen AK, Martinez-Hurtado JL, Garcia-Melendrez A, da Cruz Vasconcellos F, Lowe CR (2014). A smartphone algorithm with inter-phone repeatability for the analysis of colorimetric tests. Sens. Actuators B Chem..

[44] Koh A (2016). A soft, wearable microfluidic device for the capture, storage, and colorimetric sensing of sweat. Sci. Transl. Med..

[45] Sanka R, Lippai J, Samarasekera D, Nemsick S, Densmore D (2019). 3DμF—interactive design environment for continuous flow microfluidic devices. Sci. Rep..

[46] Kong DS (2017). Open-source, community-driven microfluidics with metafluidics. Nat. Biotechnol..

[47] Cantrell K, Erenas MM, de Orbe-Payá I, Capitán-Vallvey LF (2010). Use of the hue parameter of the hue, saturation, value color space as a quantitative analytical parameter for bitonal optical sensors. Anal. Chem..

[48] Lathwal S, Sikes HD (2016). Assessment of colorimetric amplification methods in a paper-based immunoassay for diagnosis of malaria. Lab Chip.

[49] Shen L, Hagen JA, Papautsky I (2012). Point-of-care colorimetric detection with a smartphone. Lab Chip.

[50] Welcome to Python.org. https://www.python.org/ (Accessed August 20, 2019).

[51] OpenCV. https://opencv.org/ (Accessed August 20, 2019).

[52] van der Walt S, Colbert SC, Varoquaux G (2011). The NumPy array: a structure for efficient numerical computation. Comput. Sci. Eng..

[53] tkinter—Python interface to Tcl/Tk. https://docs.python.org/3/library/tkinter.html (Accessed August 20, 2019).

[54] Mace Lab GitHub Repository for ColorScan. https://github.com/MaceLab/ColorScan.

[55] Suzuki S, Abe K (1985). Topological structural analysis of digitized binary images by border following. Comput. Vis. Graph. Image Process..

[56] Hu M-K (1962). Visual pattern recognition by moment invariants. IRE Trans. Inf. Theory.

[57] Meredith NA, Volckens J, Henry CS (2017). Paper-based microfluidics for experimental design: screening masking agents for simultaneous determination of Mn(II) and Co(II). Anal. Methods.

[58] Reading and Writing Images—OpenCV 3.0.0-dev documentation. https://docs.opencv.org/3.0-beta/modules/imgcodecs/doc/reading_and_writing_images.html#imread (Accessed August 15, 2019).

