# Supplementary material for: Open software platform for automated analysis of paper-based microfluidic devices
Source: Sci Rep. 2020 Jul 9;10:11284. doi: 10.1038/s41598-020-67639-6 (PMC7347888; doi:10.1038/s41598-020-67639-6)
Supplement: Supplementary file 1 — Supplementary file1 (PDF 3532 kb) [file 41598_2020_67639_MOESM1_ESM.pdf]

**Materials and Methods**

# Open Software Platform for Automated Analysis of Paper-Based Microfluidic Devices

Rayleigh W. Parker,[†] Daniel J. Wilson,[†] and Charles R. Mace*

Department of Chemistry, Tufts University, 62 Talbot Avenue, Medford, MA 02155

† these authors contributed equally

*Corresponding author: charles.mace@tufts.edu

Pages: 28

Figures: 18

Tables: 5

Contents: Design and fabrication details for 6 paper-based architectures used to demonstrate the capabilities of our automated image analysis software, ColorScan, presented in the order that they appear in the *User Guide* document. Details related to performance comparison of ColorScan and ImageJ in RGB, HSV, and CIELAB color spaces.

**Dye-Filled Devices**

All dye-filled devices used to demonstrate our image analysis software were prepared from wax-printed layers of Whatman 1 chromatography paper backed with double-sided adhesive (FLEXcon company) and patterned with punched holes for layer alignment.[1] The solutions of dye stored within the hydrophilic layer geometries of these devices are described in **Table S1**.

**Table S1.** Dye solutions used during fabrication of ColorScan test devices.

| solution color | concentration (mM) | dye |
|---|---|---|
| dark red | 10 | allura red |
| light red | 1 | allura red |
| dark green | 10 | tartrazine |
| | 1 | erioglaucine |
| light green | 1 | tartrazine |
| | 0.1 | erioglaucine |
| dark blue | 10 | erioglaucine |
| light blue | 1 | erioglaucine |

**ColorScan Interface Tutorial Image**

Device image. “sampleimage.jpg” (resolution: 300 dpi)

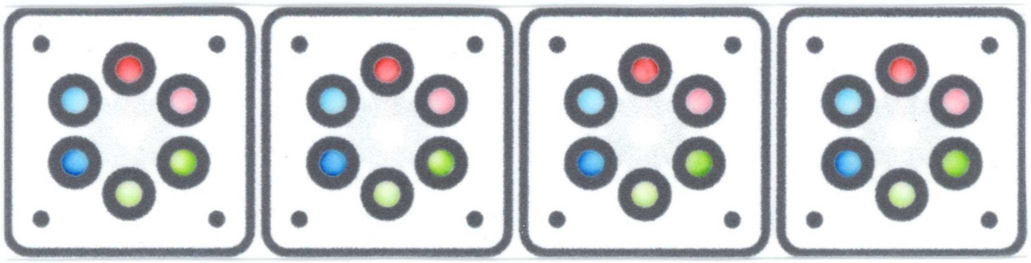

**Figure S1.** Scanned image of dye-filled output zones in three-layered paper-based microfluidic devices used to demonstrate how to perform average pixel intensity measurements using ColorScan. Each device border is approximately 1.5 x 1.5 in.

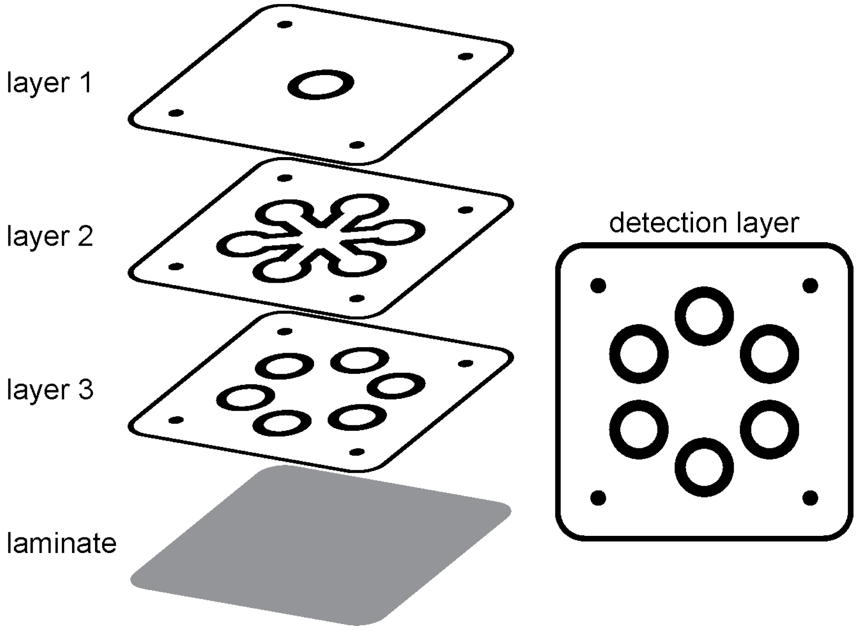


**Figure S2.** Paper-based microfluidic device design used to generate "sampleimage.jpg" image. Each layer is approximately 1.5 x 1.5 in.

Device design. This device was composed of three paper layers sealed with a transparent laminate layer (Fellowes) attached to the final paper layer. The first layer of the device accepts applied sample fluid. The second layer of the device distributes sample fluid to stored dyes, which are rehydrated and transported to the detection zones in the third layer of the device.

Reagent storage, fabrication, and device operation. To store dyes within this device, 0.3 μL of each solution shown in **Table S1** was applied to the center of a sample distribution channel in layer 2. This process was repeated four times, with an 8-minute drying step at 65 °C after each solution application. After reagent storage steps were completed, devices were assembled by removing patterned alignment holes using a leather punch, assembling paper layers over a light box, and sealing the assembly with a transparent film by lamination.[1] Each device was run with 20 μL of deionized water and scanned (Epson V600 Photo) 8 minutes after sample addition was completed.

**Histogram Analysis Tutorial Image**

Device image. “sampleimage1.jpg” (resolution: 800 dpi, depicted in **Figure 1**, **Figure 2**)

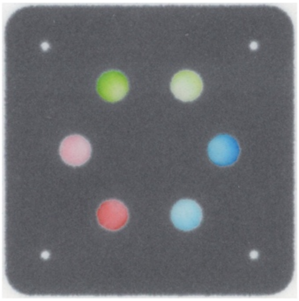

**Figure S3.** Scanned image of dye-filled output zones in a three-layered paper-based microfluidic device used to demonstrate how to perform histogram analyses using ColorScan. Device border is approximately 1.5 x 1.5 in.

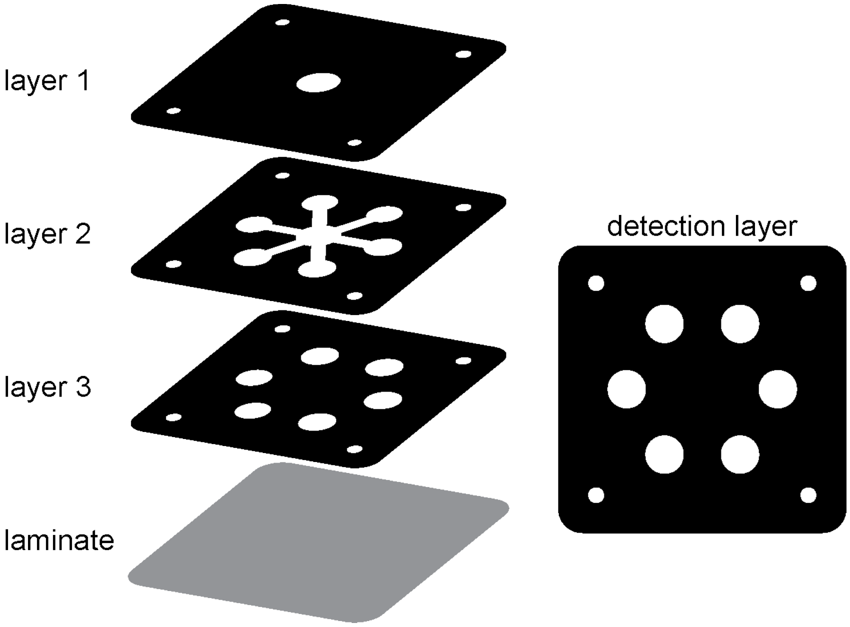


**Figure S4.** Paper-based microfluidic device design used to generate "sampleimage1.jpg" image. Each layer is approximately 1.5 x 1.5 in.

Device design. The dimensions of this device are the same as the one shown in **Figure S1**–**Figure S2**, but in this design the layer area surrounding patterned device features is filled with black wax. Alignment hole markings were formed using un-patterned paper.

Reagent storage and fabrication. To store dyes within this device, 0.3 μL of each solution shown in **Table S1** was applied to the center of a sample distribution channel in layer 2. This process was repeated four times, with a 5-minute drying step at 65 °C after each solution application. After reagent storage steps were completed, devices were assembled by removing patterned alignment holes using a leather punch, assembling paper layers over a light box, and sealing the assembly with a transparent film by lamination.[1] Each device was run with 20 μL of deionized water and scanned (Epson V600 Photo) 5 minutes after sample addition was completed.

**Zone Refinement Tutorial Image**

Device image. “refinezones.jpg” (resolution: 800 dpi)

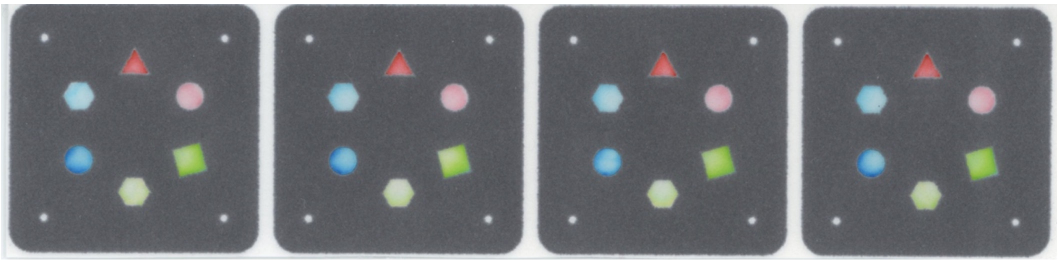

**Figure S5.** Scanned image of dye-filled output zones in a three-layered paper-based microfluidic device used to demonstrate the “circle” and “polygon” modes of the Refine Zone window in ColorScan. Each device border is approximately 1.5 x 1.5 in.

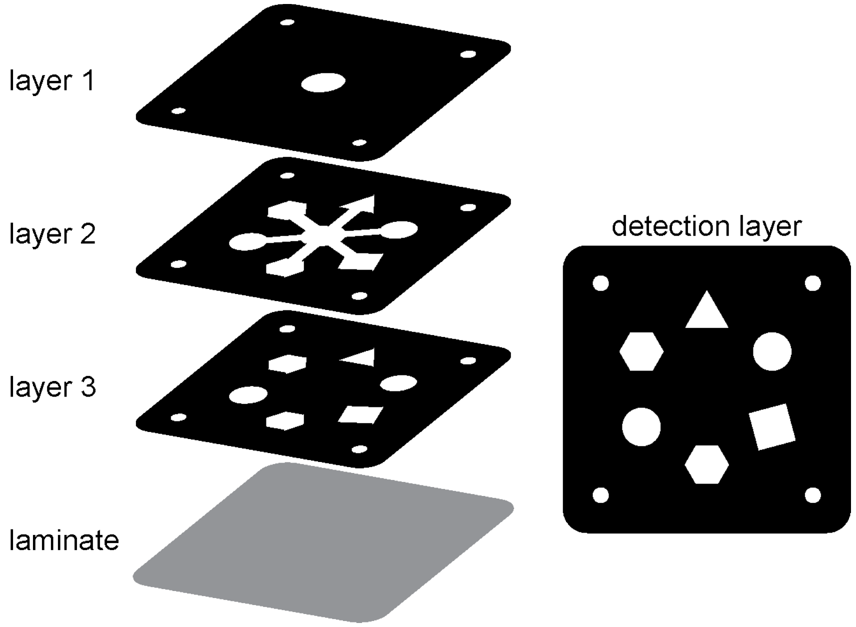


**Figure S6.** Paper-based microfluidic device design used to generate "refinezones.jpg" image. Each layer is approximately 1.5 x 1.5 in.

Device design. The dimensions of this device are the same as the one shown in **Figure S3**, but in this design the 4 circular output zones were replaced with 2 hexagonal output zones, 1 triangular output zone, and 1 square output zone. Alignment hole markings were formed using un-patterned paper.

Reagent storage and fabrication. To store dyes within this device, 0.3 μL of each solution shown in **Table S1** was applied to the center of a sample distribution channel in layer 2. This process was repeated four times, with a 5-minute drying step at 65 °C after each solution application. After reagent storage steps were completed, devices were assembled by removing patterned alignment holes using a leather punch, assembling paper layers over a light box, and sealing the assembly with a transparent film by lamination.[1] Each device was run with 20 μL of deionized water and scanned (Epson V600 Photo) 10 minutes after sample addition was completed.

**Lateral Flow Immunoassay Device**

Device image. “lateralflowstrip.jpg” (resolution: 800 dpi)

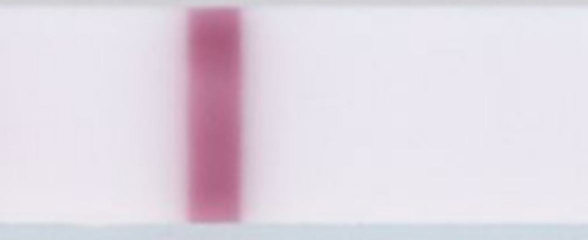

**Figure S7.** Scanned image of the control line of a commercial lateral flow test used to demonstrate the “rectangle” mode of the Refine Zone window in the ColorScan software. Image scaled to 10x scanned size for visualization.

Uncropped device image.

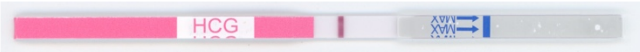


**Figure S8.** Original scanned image of lateral flow test. Image is not scaled.

Device design. We purchased this commercial pregnancy test from Amazon.com (ClinicalGuard HCG Pregnancy Test Strips, item # B007VT30C8, $0.32 per device in September 2019).

Device operation. We ran this device using 1X PBS. Following the manufacturer’s instructions, we immersed the sample application end of the test strip into our sample solution (to just under the MAX line), laid the strip flat, and scanned the device (Epson V600 Photo) after 5 minutes.

**Selecting Colorimetric Signal at the Ends of Paper Channels**

Device image. “channelends.jpg” (resolution: 800 dpi, depicted in **Figure 3**)

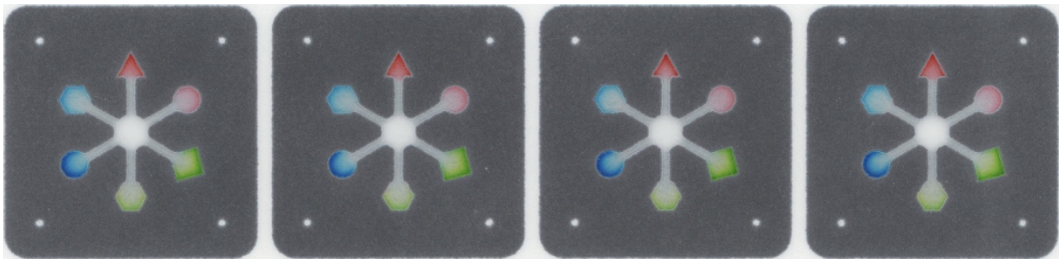

**Figure S9.** Scanned image of dye-filled output zones in a two-layered paper-based microfluidic device used to demonstrate selection of colorimetric signal at the ends of paper channels using the Refine Zone window in the ColorScan software. Each device border is approximately 1.5 x 1.5 in.

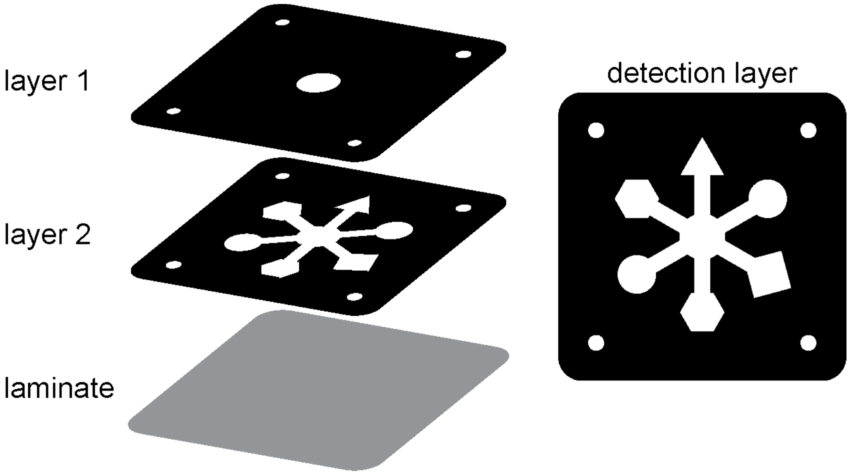


**Figure S10.** Paper-based microfluidic device design used to generate “channelends.jpg” image. Each layer is approximately 1.5 x 1.5 in.

Device design. The dimensions of this device are the same as the one shown in **Figure S5**, but this device contains only two paper layers, meaning that colorimetric signal is contained at the ends of the fluid distribution channels. Alignment hole markings were formed using un-patterned paper.

Reagent storage and fabrication. To store dyes within this device, the solutions shown in **Table S1** were applied to the ends of the sample distribution channels in layer 2 of the device. Each different zone shape required a different volume to fill: the triangular zone required 0.4 μL, the circular zones required 0.75 μL each, the square zone required 1 μL, and the hexagonal zones required 0.85 μL each. This device layer was dried for 8 minutes at 65 °C after the dye solutions were applied. Patterned alignment hole markers were then removed using a leather punch and paper layers were assembled over a light box, then the device assembly was sealed with a transparent film by lamination.[1] Each device was run with 20 μL of deionized water, which rehydrated the dye stored at the end of each channel in layer 2, and scanned (Epson V600 Photo) 8 minutes after sample application was completed.

**ColorScan and ImageJ Comparison Devices**

Device image. “comparison.jpg” (resolution: 800 dpi, depicted in **Figure 4**)

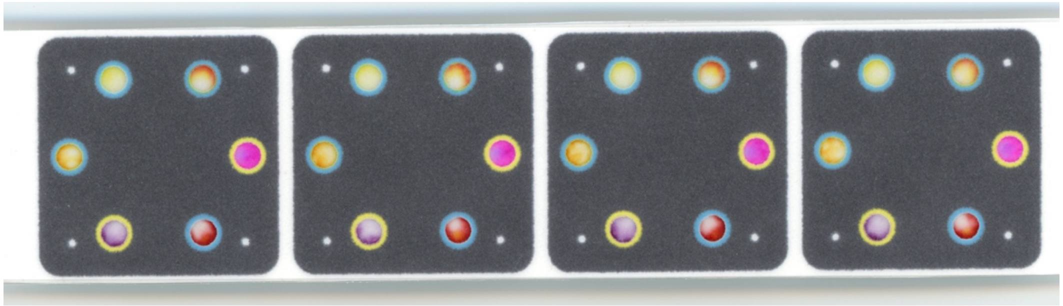

**Figure S11.** Scanned image of output zones in a four-layered paper-based microfluidic device designed to form six different colorimetric signals. This image was used to compare the performance of ColorScan and ImageJ. Each device border is approximately 1.5 x 1.5 in.

Image of device before sample application. (resolution: 800 dpi)

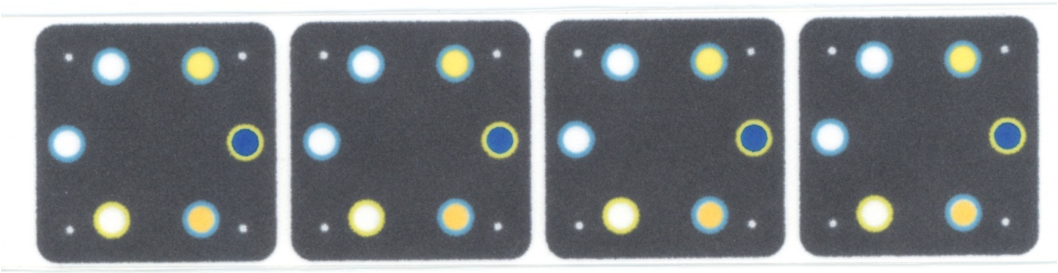

**Figure S12.** Scanned image of the device shown in **Figure S11** before colorimetric assays were initiated by application of water to the sample application layer. Each device border is approximately 1.5 x 1.5 in.

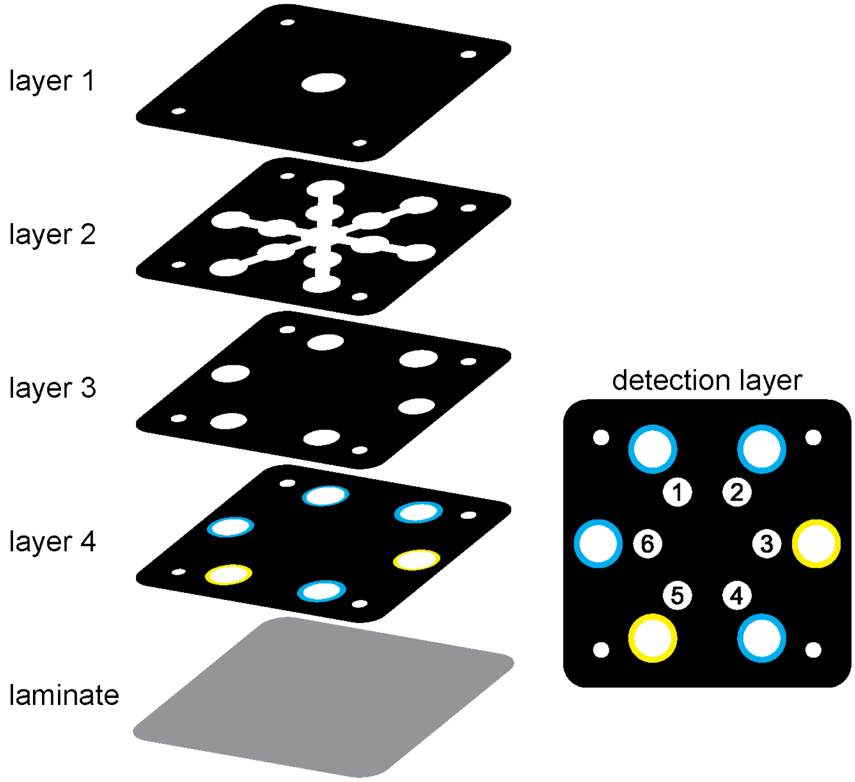


**Figure S13.** Paper-based microfluidic device design used to generate "comparison.jpg" image. Each layer is approximately 1.5 x 1.5 in. The output zone numbers depicted on the detection layer match the "position" column in **Table S2**.

Device design. This device is composed of four paper layers and sealed by a layer of transparent laminate. The first layer of this device accepts applied sample fluid. Reagents required to complete colorimetric reactions are stored in layer 2, layer 3, and layer 4 of the device. Each fluid distribution channel in layer 2 contains two reagent storage zones, the second of which connects to the reagent storage zones in layer 3. These zones are aligned with the output zones in layer 4, each of which is surrounded by a printed contrast ring shown in the scanned image above. As a sample of deionized water fills the device, stored reagents are rehydrated and mixed to produced colorimetric signal in each output zone.

Reagent storage and fabrication. The solutions used to store reagents within the paper layers of these devices are detailed in **Table S2**. All reagent storage steps were performed by pipetting 1 µL of solution into its respective circular zone. Each treated device layer was dried for at least 5 minutes at 65 °C. Next, patterned alignment hole markers were removed using a leather punch, then paper layers were assembled over a light box and sealed with a transparent film by lamination.[1]

Device operation. Each device was run using 45 µL of deionized water and scanned 10 minutes after sample application. In output zone 1, yellow signal is formed by a reaction between the sulfhydryl group of cysteine and Ellman's reagent (5,5′-Dithiobis(2-nitrobenzoic acid)). In output zone 2, an orange signal is formed when stored phenol red is rehydrated by pH 7 TRIS buffer. In output zone 3, methyl green is converted to a colorless product by reaction with sulfite to reveal the pink color of rehydrated Rhodamine B.[2] In output zone 4, copper(II) forms a red colored complex with 4-(2-pyridylazo)resorcinol (PAR), a colorimetric indicator for a variety of cations.[3] In output zone 5, iron(II) produced by reduction of iron(III) using ascorbic acid is chelated by Ferrozine (3-(2-pyridyl)-5,6-diphenyl-1,2,4-triazine-p,p'-disulfonic acid) to produce purple signal.[4] In output zone 6, molybdate ion forms a yellow-orange colored complex with Tiron (1,2-dihydroxybenzene-3,5-disulfonic acid), which has previously been used to quantify mixing in fluidic systems.[5]

**Table S2.** Reagent solutions used during paper-based device fabrication.

| layer | position | solute | concentration | solvent |
|---|---|---|---|---|
| 2 (inner) | 1 | cysteine | 5 mM | water |
| | 2 | - | - | 200 mM TRIS-HCl buffer pH 7.1 |
| | 3 | sodium sulfite | 1 M | water |
| | 4 | cobalt(II) nitrate | 20 mM | water |
| | 5 | iron(III) chloride | 10 mM | water |
| | 6 | sodium molybdate(VI) | 50 mM | water |
| 2 (outer) | 1 | cysteine | 5 mM | water |
| | 2 | - | - | 200 mM TRIS-HCl buffer pH 7.1 |
| | 3 | sodium sulfite | 1 M | water |
| | 4 | sodium pyrophosphate | 100 mM | 1 M glycine buffer pH 9.7 |
| | 5 | iron(III) chloride | 10 mM | water |
| | 6 | sodium molybdate(VI) | 50 mM | water |
| 3 | 1 | cysteine | 5 mM | water |
| | 2 | - | - | 200 mM TRIS-HCl buffer pH 7.1 |
| | 3 | sodium sulfite | 1 M | water |
| | 4 | sodium pyrophosphate | 100 mM | 1 M glycine buffer pH 9.7 |
| | 5 | L-ascorbic acid | 100 mM | water |
| | 6 | sodium molybdate(VI) | 50 mM | water |
| 4 | 1 | 5,5′-dithiobis(2-nitrobenzoic acid) (Ellman's reagent) | 5 mM | 100 mM phosphate buffer pH 7.2, 0.1 mM EDTA |
| | 2 | phenol red | saturated (<10 mM) | water |
| | 3 | methyl green<br>rhodamine B | 6 mM<br>4 mM | water |
| | 4 | 4-(2-pyridylazo) resorcinol (PAR) | 5 mM | 125 mM borate buffer pH 10.0 |
| | 5 | 3-(2-pyridyl)-5,6-diphenyl-1,2,4-triazine-p,p'-disulfonic acid (Ferrozine) | 50 mM | water |
| | 6 | 1,2-dihydroxybenzene-3,5-disulfonic acid (Tiron) | 100 mM | water |

## ColorScan Performance Evaluations

RGB color intensity analysis in ImageJ.

When we manually analyzed the image shown in **Figure S11** using ImageJ, we used a circular region of interest (125 x 125 pixels, ImageJ area: 12281). This region was manually centered on each output zone in the image and measurements were performed using the "RGB Measure" plugin. Results were manually copied and pasted into a previously formatted Microsoft Excel spreadsheet. These measurement steps took approximately 12 minutes to complete.

Image processing in Photoshop.

To obtain a cropped image of each output zone shown in **Figure S11**, we made a 0.275 x 0.275 in. square selection around each output zone contrast ring in Adobe Photoshop. In the right-click menu, we selected "Layer Via Copy" for each selection. After all output zones were copied to individual layers, we selected all of these layers at once and used "Quick Export as PNG" to obtain an image file for each output zone. These image processing steps took approximately 12 minutes to complete, resulting in a total time of 24 minutes for our manual evaluation.

Image analysis and processing in ColorScan.

Analysis of the image shown in **Figure S11** using ColorScan took approximately 2 minutes and automatically provided organized measurement results and cropped images of each device output zone. During this analysis, we were not able to select the exact same analysis area used for our ImageJ measurements. The 125 x 125 pixel circular analysis area (radius: 62.5 pixels) showed a calculated area of 12281 pixels in ImageJ, while the circular region that we selected using ColorScan showed a calculated area of 11681 pixels in the Refine Zone window. Note

that neither of these pixel areas exactly match the area of a circle corresponding to the given radius, because pixelated shapes are by nature approximations to the continuous case. As shown in **Figure S14**, both of these analysis regions appear to appear to effectively encompass the signal in each output zone. Accordingly, we do not expect that his discrepancy should impact the outcome of our performance comparison between ColorScan and ImageJ.

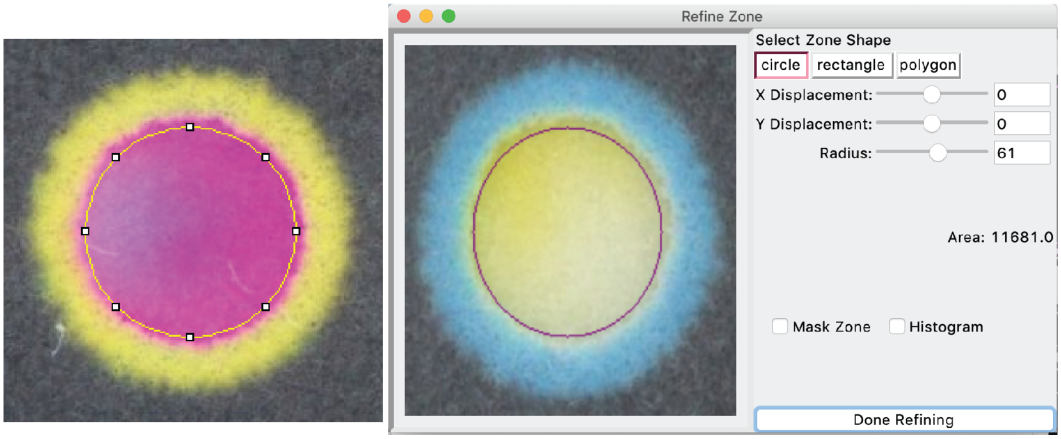


**Figure S14.** Selection of analysis geometries. Analysis circles from ImageJ (left) and ColorScan (right). The image within the ColorScan Refine Zone window appears slightly skewed due to the aspect ratio of the window, but the analysis geometry displayed in red is a true circle.

The measurement results provided by ColorScan were organized according to the numbers shown in **Figure S15** and did not match the analysis order we followed during our ImageJ analysis protocol. We manually reorganized these results in Microsoft Excel to facilitate comparison to the results obtained using ImageJ.

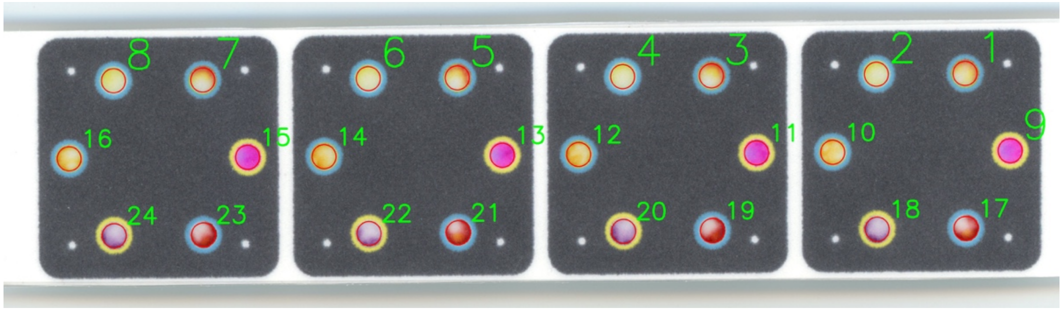


**Figure S15.** ColorScan output zone and measurement result numbering.

Mean pixel intensity variance in manual and automated RGB image analyses.

As shown in **Table S3**, the standard deviation of mean pixel intensity for replicate output zones measured using ColorScan is comparable to that of devices analyzed using ImageJ.

**Table S3.** Standard deviations of mean RGB pixel intensity in replicate output zones of 4 paper-based microfluidic devices (**Figure S11**).

| | **Spot #** | **1** | **2** | **3** | **4** | **5** | **6** |
|---|---|---|---|---|---|---|---|
| | | **replicate output zone std. dev.** | | | | | |
| **ImageJ** | Red | 1.3 | 3.5 | 3.4 | 4.8 | 3.7 | 3.7 |
| | Green | 0.9 | 4.1 | 6.2 | 7.9 | 6.2 | 5.6 |
| | Blue | 4.7 | 1.8 | 5.4 | 9.5 | 3.4 | 8.7 |
| **ColorScan** | Red | 1.2 | 3.5 | 3.4 | 4.5 | 3.5 | 3.6 |
| | Green | 0.8 | 4.7 | 6.4 | 7.3 | 6.0 | 5.4 |
| | Blue | 5.2 | 1.8 | 5.5 | 9.2 | 3.2 | 8.5 |

Comparison of HSV and CIELAB measurements performed using ImageJ and ColorScan.

*Color Space Conversions for Manual Analysis in ImageJ*

To facilitate comparison of the accuracy and consistency of measurements performed in the HSV and CIELAB color spaces using ImageJ and ColorScan, we converted the RGB image shown in **Figure S11** to these color spaces using ImageJ. To convert to the HSV color space, we selected Type > HSB Stack from the "Image" menu. To convert to the CIELAB color space, we used the Color Transformer plugin[6] and selected the "Lab" option. The greyscale images provided by each conversion are shown in **Figure S16** and **Figure S17**.

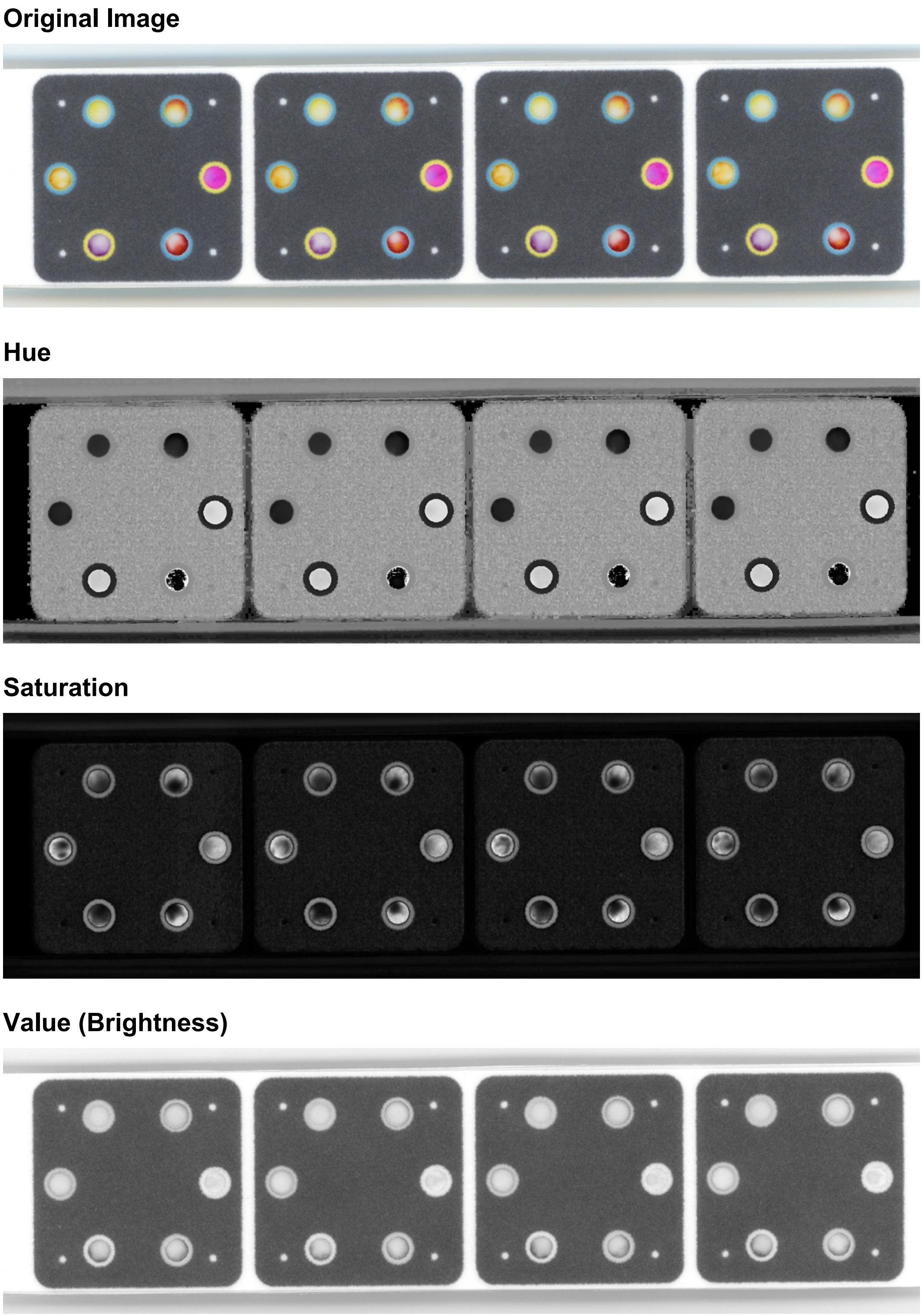


**Figure S16.** Conversion of scanned RGB image to HSV color space using ImageJ.

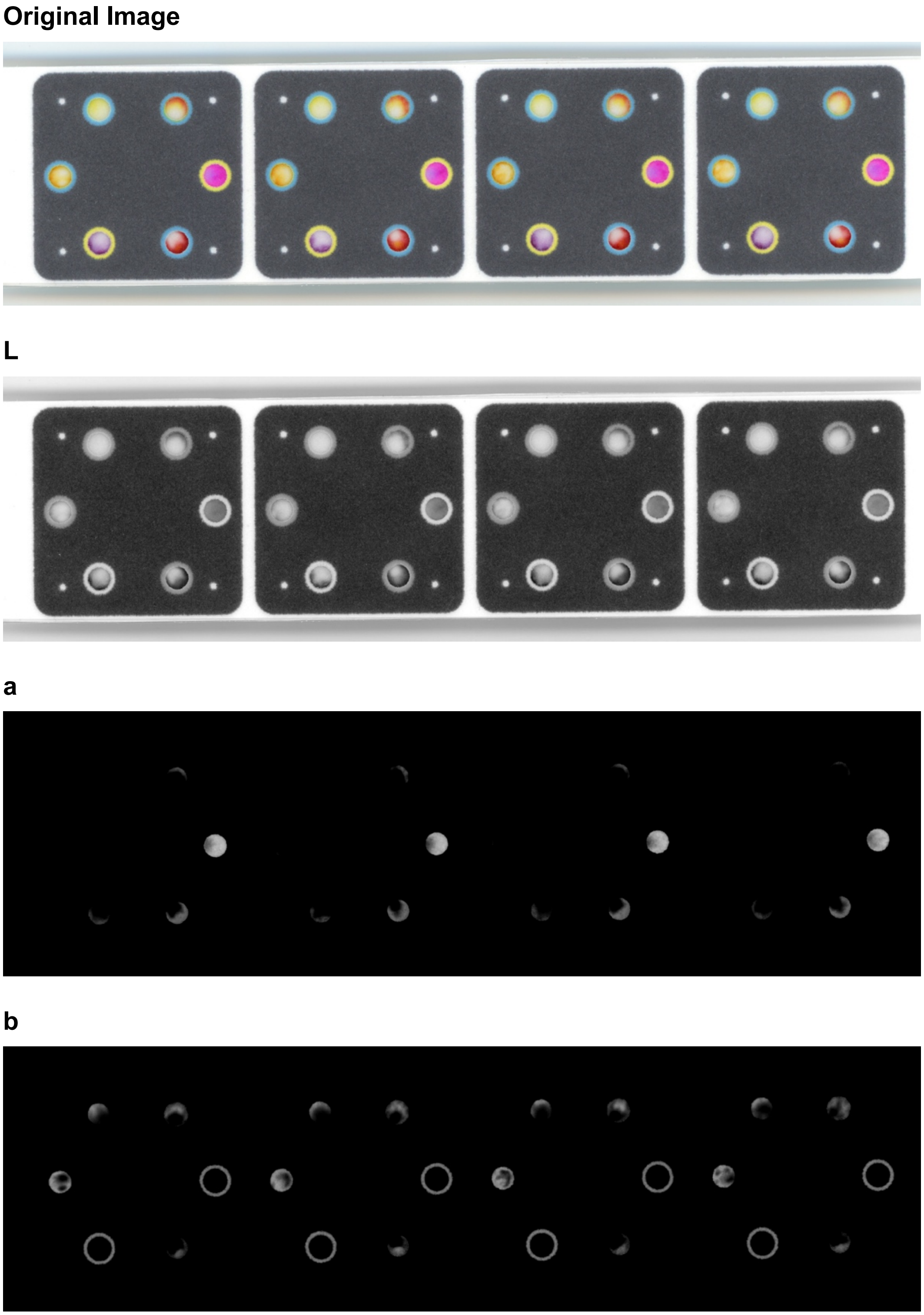


**Figure S17.** Conversion of scanned RGB image to CIELAB color space using ImageJ.

*ImageJ Analysis and Comparison to ColorScan Results*

Following the same approach used in our RGB measurement performance comparison, we used a 125 x 125 pixel circular region of interest to manually analyze colorimetric signals in the HSV and CIELAB color spaces. Signals from each zone were acquired from each converted image using the Measure tool under the “Analyze” menu. ImageJ presented Hue, Saturation, and Brightness measurements on a scale of 0 to 255, but ColorScan uses a different scaling system in which Hue is presented in degrees (ranging from 0 to 360) and both Saturation and Value (Brightness) are both presented as decimal values (ranging from 0 to 1). To scale Hue measurements acquired using ImageJ to match the format of ColorScan results, we divided ImageJ Hue measurements by 255, then multiplied them by 360. For Saturation and Value measurements, we divided ImageJ results by 255. CIELAB measurements acquired using ImageJ did not require scaling for comparison to ColorScan results. ImageJ results were compared to HSV and CIELAB results that were acquired using ColorScan at the same time as the previously described measurements for our RGB performance comparison.

Generally, both HSV and CIELAB measurements performed using ColorScan compared favorably to paired measurements performed manually using ImageJ (**Table S4, Table S5**). However, there were some discrepancies observed for zones that contained signal that formed or was distributed heterogeneously throughout the detection area. For example, when our original scanned image of device detection zones (**Figure S11**) was converted to the HSV color space, spot #4 (**Figure S16**) contained a bimodal distribution of pixel intensities in the greyscale image representing Hue (**Figure S16**). We noticed that the measured Hue of these spots differed considerably (~32%) between ColorScan and ImageJ and that measurement consistency for these detection zones suffered in both programs. We attribute this to (i) differences in analysis region placement that occurred during manual analysis using ImageJ

and (ii) inconsistencies in the total numbers of black and white pixels across replicate output in the greyscale image representing Hue. Both of these factors caused pixels that drastically differ in intensity (i.e., opposite ends of our measurement range) to be included or excluded during analysis. The heterogeneity of color distribution not only makes quantitative analysis of device images challenging but would also likely preclude interpretation of these signals by visual inspection.

Additionally, mean measured values differed (~7–19%) when the color space conversion caused signal-containing pixels to be difficult to distinguish from (i) the pixels that were indicative of the spot border (e.g., “a” measurements for spot #2 in the CIELAB color space) or (ii) pixels that were outside of the detection zone (e.g., “a” measurements for spot #6 in the CIELAB color space). In both of these examples, heterogenous development or presentation of colorimetric signal confounds device interpretation by both image analysis and visual inspection. We show these cases here to demonstrate the kinds of “good signals” and “bad signals” that one could encounter in assay development.

In the majority of cases evaluated here, ColorScan provides accurate measurement results in comparison to ImageJ. These results highlight the utility of ColorScan for automatically determining which color spaces or channels best support measurement of different kinds of colorimetric signals. In all image analysis circumstances—whether ColorScan, ImageJ, or another program is used to quantify colorimetric signal—spot quality and homogenous formation of color within a device detection zone will be the main driving force behind the accurate and consistent measurement results.

**Table S4.** Mean HSV measurements acquired using ImageJ and ColorScan. Standard deviation values calculated from four replicate measurements of each numbered output zone.

| | Spot # | 1 | 2 | 3 | 4 | 5 | 6 |
|---|---|---|---|---|---|---|---|
| | | mean measured signal | | | | | |
| **ImageJ** | H | 62.5 | 42.7 | 306.8 | 62.9 | 291.4 | 46.1 |
| | S | 0.293 | 0.402 | 0.626 | 0.505 | 0.207 | 0.532 |
| | V | 0.836 | 0.829 | 0.881 | 0.742 | 0.718 | 0.848 |
| **ColorScan** | H | 63.0 | 43.8 | 307.0 | 42.9 | 291.6 | 46.5 |
| | S | 0.290 | 0.400 | 0.627 | 0.498 | 0.205 | 0.534 |
| | V | 0.841 | 0.834 | 0.882 | 0.752 | 0.726 | 0.853 |
| **% difference** | H | 0.8 | 2.5 | 0.1 | 31.8 | 0.1 | 1.0 |
| | S | 1.1 | 0.4 | 0.2 | 1.3 | 1.1 | 0.6 |
| | V | 0.5 | 0.6 | 0.1 | 1.3 | 1.1 | 0.6 |
| | | replicate output zone std. dev. | | | | | |
| **ImageJ** | H | 0.56 | 0.91 | 0.73 | 13.06 | 0.91 | 1.19 |
| | S | 0.02 | 0.02 | 0.02 | 0.04 | 0.02 | 0.04 |
| | V | 0.00 | 0.01 | 0.01 | 0.02 | 0.01 | 0.01 |
| **ColorScan** | H | 0.60 | 0.75 | 0.71 | 9.76 | 0.82 | 1.06 |
| | S | 0.02 | 0.01 | 0.02 | 0.04 | 0.02 | 0.04 |
| | V | 0.00 | 0.01 | 0.01 | 0.02 | 0.01 | 0.01 |

**Table S5.** Mean CIELAB measurements acquired using ImageJ and ColorScan. Percent difference between ImageJ and ColorScan values was calculated using the magnitudes of mean signals. Standard deviation values calculated from four replicate measurements of each numbered output zone.

| | Spot # | 1 | 2 | 3 | 4 | 5 | 6 |
|---|---|---|---|---|---|---|---|
| | | **mean measured signal** | | | | | |
| **ImageJ** | L | 83.8 | 74.5 | 58.7 | 55.8 | 65.2 | 77.2 |
| | a | -10.0 | 6.0 | 66.6 | 31.9 | 17.5 | -0.3 |
| | b | 30.0 | 32.0 | -34.4 | 21.2 | -13.2 | 47.1 |
| **ColorScan** | L | 84.2 | 75.7 | 58.8 | 56.4 | 65.8 | 77.7 |
| | a | -9.9 | 4.9 | 66.7 | 31.8 | 17.5 | -0.2 |
| | b | 30.0 | 31.9 | -34.9 | 21.5 | -13.5 | 47.0 |
| **% difference** | L | 0.5 | 1.6 | 0.2 | 1.2 | 1.0 | 0.7 |
| | a | 1.0 | 18.7 | 0.2 | 0.2 | 0.3 | 7.5 |
| | b | 0.2 | 0.4 | 1.4 | 1.0 | 1.9 | 0.3 |
| | | **replicate output zone std. dev.** | | | | | |
| **ImageJ** | L | 0.4 | 1.4 | 1.5 | 2.1 | 2.0 | 1.8 |
| | a | 0.6 | 2.0 | 1.4 | 1.9 | 1.6 | 1.2 |
| | b | 2.1 | 1.9 | 1.2 | 2.9 | 1.2 | 2.4 |
| **ColorScan** | L | 0.4 | 1.4 | 1.5 | 2.1 | 1.9 | 1.7 |
| | a | 0.6 | 1.7 | 1.4 | 2.1 | 1.5 | 1.2 |
| | b | 2.3 | 1.9 | 1.2 | 3.2 | 1.2 | 2.4 |

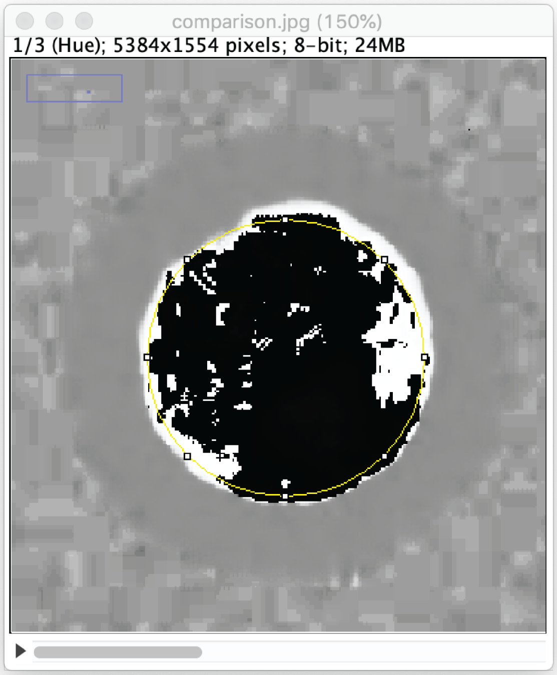


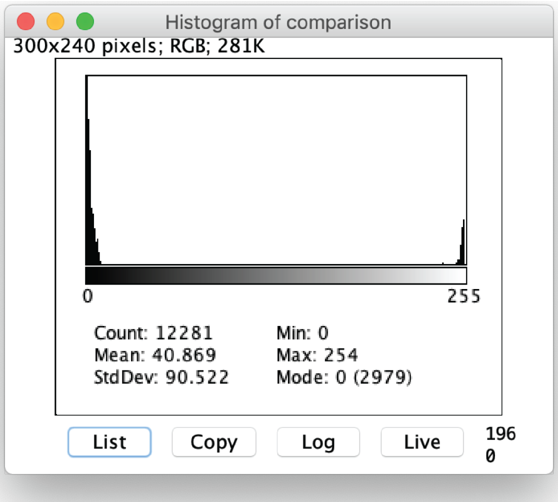


**Figure S18.** Conversion of the original image shown in **Figure 4A** to the HSV color space caused binarization of the colorimetric signal in the detection zone indicating the presence of cobalt(II) (left). When the converted greyscale image representing Hue (**Figure S16**) was measured using ImageJ, the binarized pixels provided a bimodal distribution of measured intensity (right). The measured pixel intensities shown in the histogram are presented on the same scale as measured RGB values (0 to 255) but represent the measured hue of the pixels within the selected region of interest.

## References


**1.** Fernandes, S. C., Wilson, D. J. & Mace, C. R. Fabrication of Three-dimensional Paper-based Microfluidic Devices for Immunoassays. *Journal of Visualized Experiments* **121**, e55287, https://doi.org/10.3791/55287 (2017).

**2.** Vella, S. J., *et al.* Measuring Markers of Liver Function Using a Micropatterned Paper Device Designed for Blood from a Fingerstick. *Analytical Chemistry* **84**, 2883–2891, https://doi.org/10.1021/ac203434x (2012).

**3.** Meredith, N. A., Volckens, J. & Henry, C. S. Paper-based microfluidics for experimental design: screening masking agents for simultaneous determination of Mn(II) and Co(II). *Analytical Methods* **9**, 534–540, https://doi.org/10.1039/C6AY02798A (2017).

**4.** Gibbs, C. R. Characterization and Application of FerroZine Iron Reagent as a Ferrous Iron Indicator. *Analytical Chemistry* **48**, 1197–1201, https://doi.org/10.1021/ac50002a034 (1976).

**5.** Oates, P. M.; Harvey, C. F. A colorimetric reaction to quantify fluid mixing. *Experiments in Fluids* **41**, 673–683, https://doi.org/10.1007/s00348-006-0184-z (2006).

**6.** Barilla, M. E. Color Transformer. https://imagej.nih.gov/ij/plugins/color-transforms.html (2007).
