# Supplementary material for: Open software platform for automated analysis of paper-based microfluidic devices
Source: Sci Rep. 2020 Jul 9;10:11284. doi: 10.1038/s41598-020-67639-6 (PMC7347888; doi:10.1038/s41598-020-67639-6)
Supplement: Supplementary file 2 — Supplementary file2 (PDF 4936 kb) [file 41598_2020_67639_MOESM2_ESM.pdf]

Python Installation Instructions and ColorScan User Guide

Open Software Platform for Automated Analysis of Paper-Based Microfluidic Devices

Rayleigh W. Parker,[†] Daniel J. Wilson,[†] and Charles R. Mace*

Department of Chemistry, Tufts University, 62 Talbot Avenue, Medford, MA 02155

† these authors contributed equally

*Corresponding author: charles.mace@tufts.edu

Pages: 45

I. Python Installation Instructions

The ColorScan script is written in Python 3, and relies on the Numpy, Matplotlib, TkInter, PIL (pillow), and OpenCV libraries to operate. ColorScan is not compatible with Python 2. We recommend Anaconda, a distribution of Python intended for scientific applications, for ColorScan users who are new to Python. With the exception of OpenCV, Anaconda comes with all of the libraries required to run ColorScan and its package manager, Conda, simplifies the installation of additional packages. To get Anaconda, go to www.anaconda.com/download/ and click on the download button for the appropriate Python 3 version for your operating system.

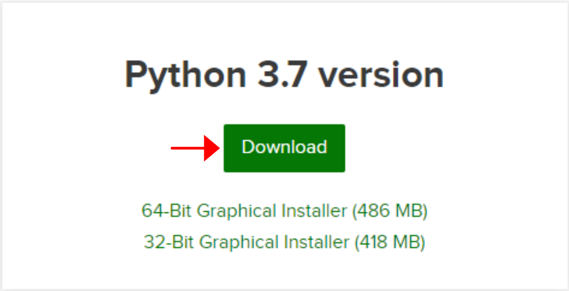

We recommend adding Anaconda to the PATH environment variable during the installation to make it your computer's default Python version. Additionally, we recommend that you do not install Microsoft VSCode during the Anaconda installation process, as it is not required to run ColorScan.

Anaconda does not include OpenCV, so this library must be installed using Terminal (Mac) or Command Prompt (Windows). A Terminal window can be accessed from a Spotlight search on Mac, and a Command Prompt window can be accessed from searching within the Start Menu on Windows.

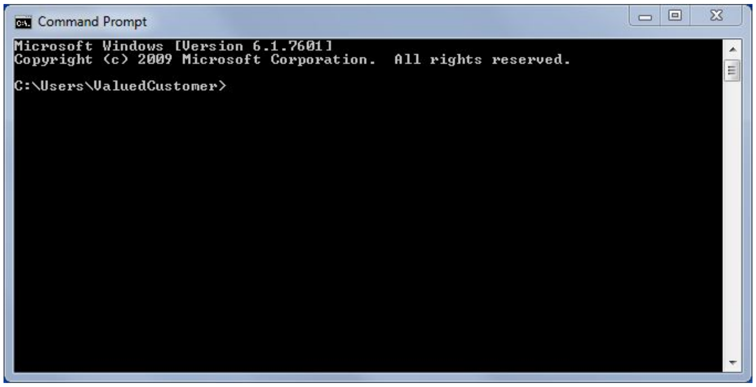

To install OpenCV, open a Terminal or Command Prompt window and run the following line:

```
conda install -c menpo opencv
```

The line can be run by typing the above text, exactly as written, into the console window and pressing the Enter key. If this installation approach fails, or if you are not using Anaconda, OpenCV can be installed using Python’s Pip package installer. To install OpenCV using Pip, run the following line:

```
pip install opencv-python
```

Once the requisite packages are installed, the ColorScan script can be run using Python3. Our ColorScan script file ("ColorScan.py") and all of the example images of paper-based devices shown in this tutorial are available for download at:

https://github.com/MaceLab/ColorScan

II. ColorScan User Guide

Running the Python Script

There are many options for editing and running the Python Script. We prefer IDLE, which is included in the Anaconda installation and is specifically designed for use with Python. To open IDLE, open a Terminal or Command Prompt window and run:

```
idle
```

Note: If your computer has a previous Python 2 installation, you may need to specify that you want to run Python 3 by typing:

```
idle3
```

Running this command should open the Python 3 Shell.

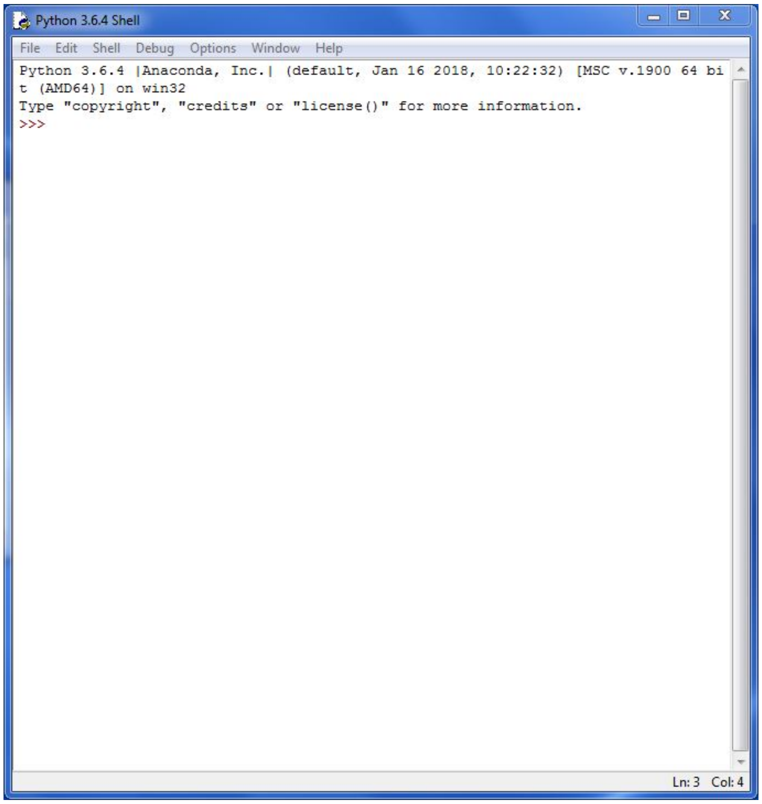

From the Shell window, use File > Open… to select the desired Python script. To open ColorScan, select "ColorScan.py" from wherever it is saved to your computer. In this demonstration, the "ColorScan.py" script file is saved in a folder called "ColorScan" on the Desktop.

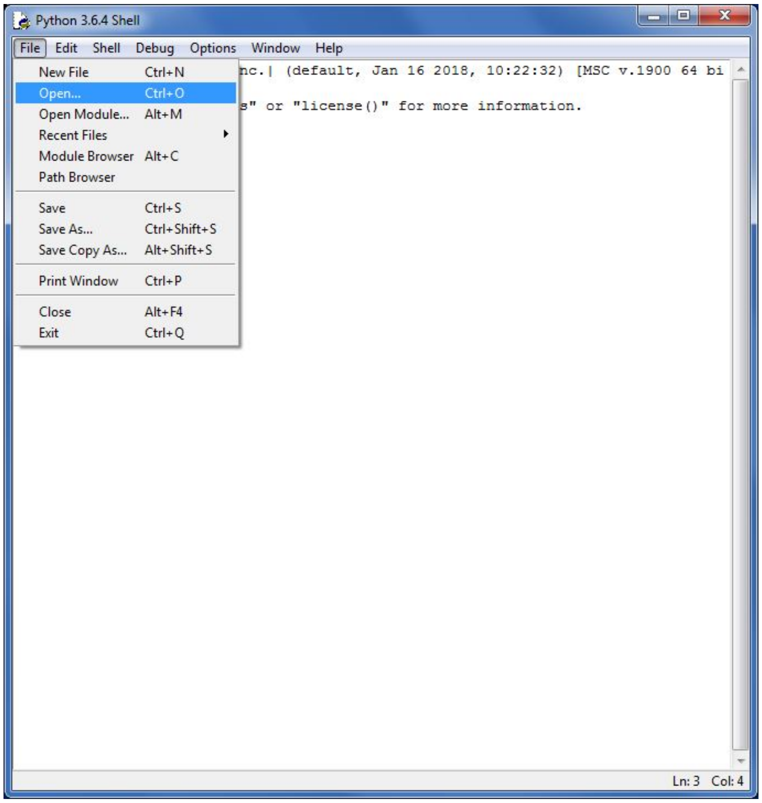

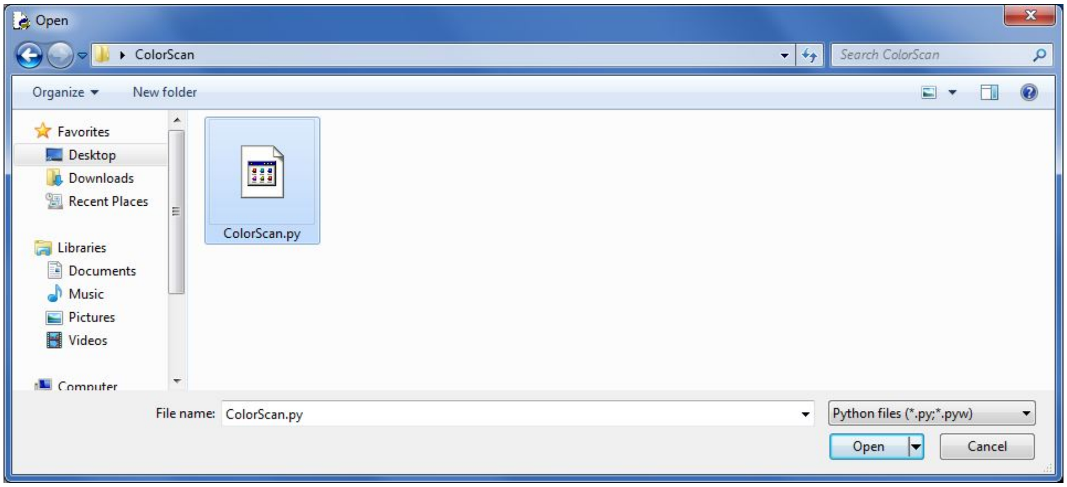

Using the ColorScan Interface

Device Image: “sampleimage.jpg” (resolution: 300 dpi)

After opening the script file, use Run > Run Module (or press the F5 key) to open the ColorScan interface. The interface should appear as a small window, which will expand to fit the image selected for analysis. For the ColorScan icon (not shown in this tutorial) to be displayed in the top left corner of the user interface windows (on compatible operating systems), the “ColorScanIcon.ico” file must be saved to the same location as the “ColorScan.py” script file.

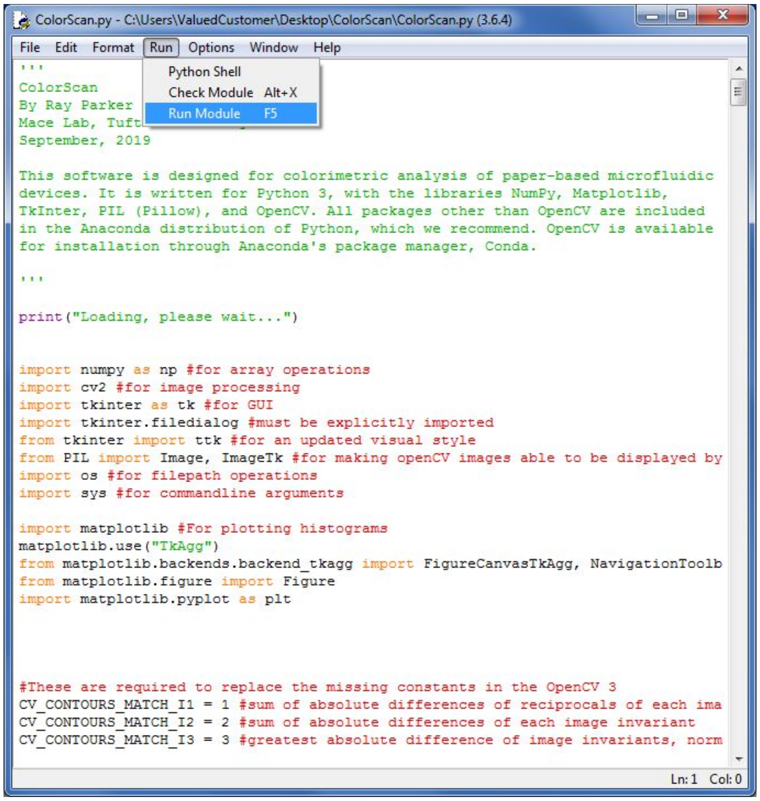

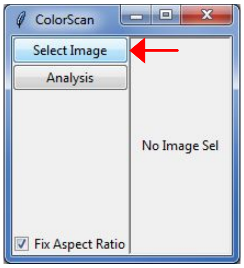

Within the interface, clicking the “Select Image” button will open a file browser. Navigate to the location where the desired image file (ex. “sampleimage.jpg”) is saved. Select the file and click “Open”.

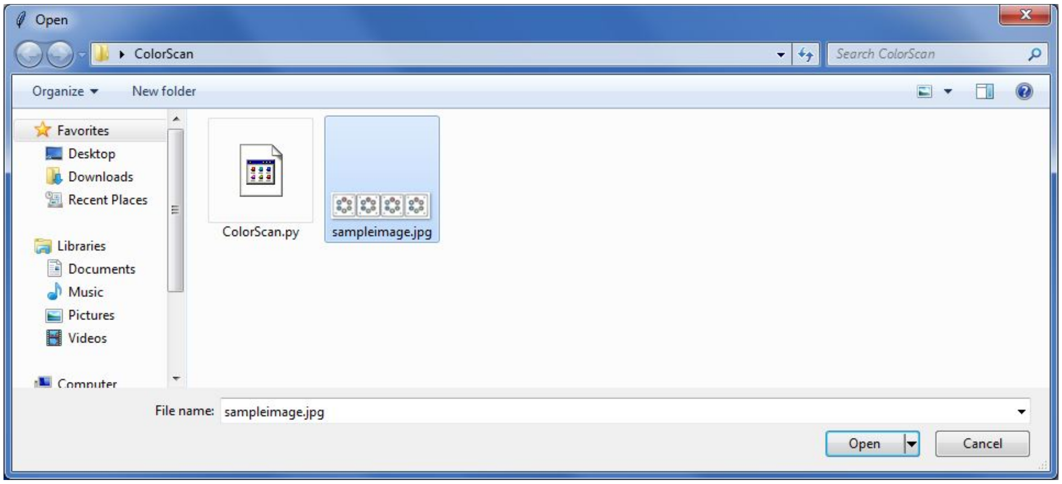

The image should be displayed within the main ColorScan window. The image we have chosen for demonstrating ColorScan shows four multilayered paper-based devices, each comprising six radially distributed circular zones filled with solutions of red, green, and blue dyes. Details of device fabrication are available in the **Materials and Methods** document. In the interface window, the “Fix Aspect Ratio” box may be checked to maintain the aspect ratio of the original image when the interface window is resized. The size and aspect ratio of the displayed image do not affect the data contained within the image or any part of the analysis.

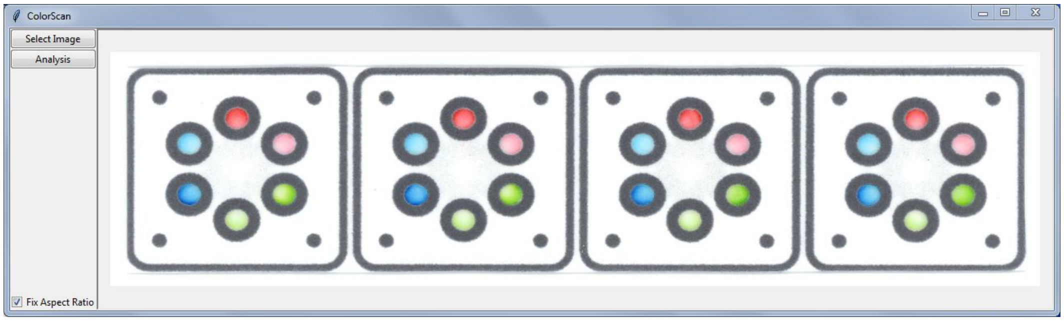

Clicking on the “Analysis” button will open a separate Analysis Menu window. The buttons, sliders, and fields within this window are arranged from top to bottom to follow the sequence in which the analysis steps should be performed. The features of this window that correspond to the later steps of the analysis process are not clickable when the window is first opened, but later become accessible in a stepwise manner to ensure that they are used in the correct order.

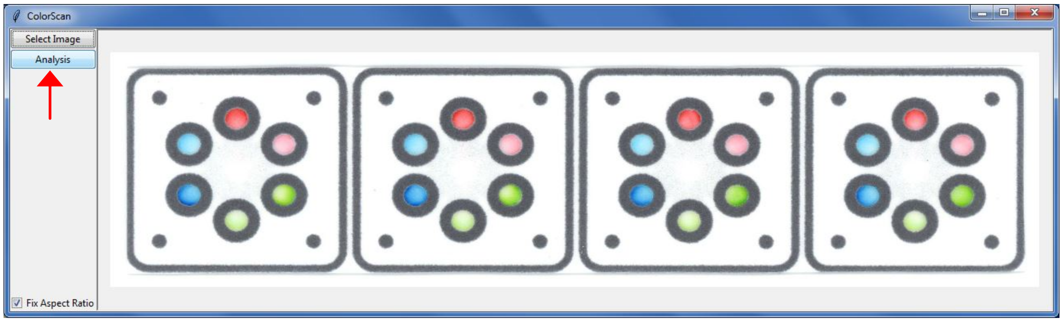

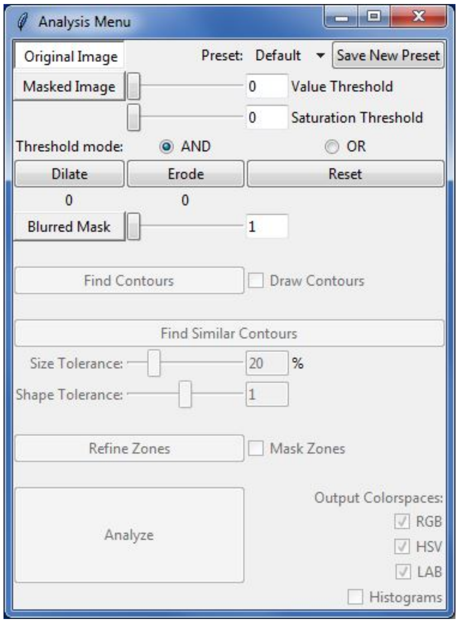

The first step in the analysis process is to mask the image using the Value (i.e., the Value component of the HSV color space) and Saturation Thresholds. Clicking the “Masked Image” button will display the masked image as these values are adjusted. Each threshold can be changed using its slider, the keyboard arrows, or by typing a specific value into its text field. The Value Threshold should be increased until the hydrophobic borders of the device geometry are clearly defined, and then the Saturation Threshold can be increased until the white areas of the binarized image approximately indicate areas of colorimetric signal within the device. Using the “Original Image” button, the user may switch back to viewing the original image to ensure accurate selection of device output zones.

Value Threshold:

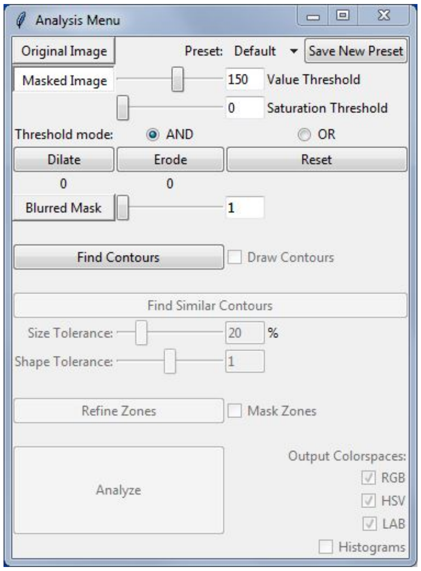

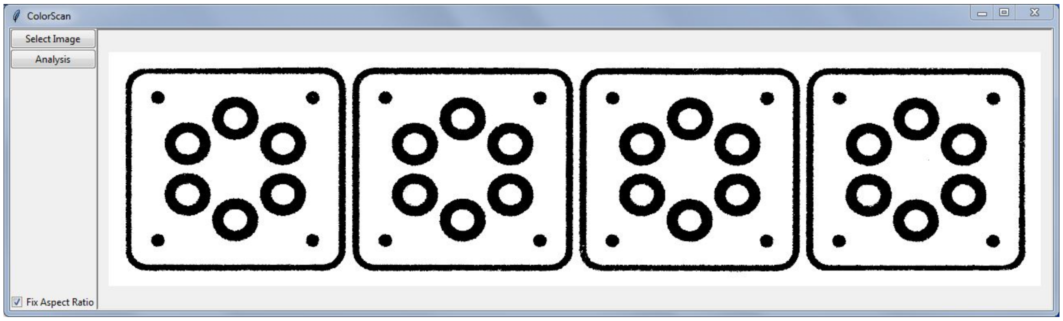

Saturation Threshold:

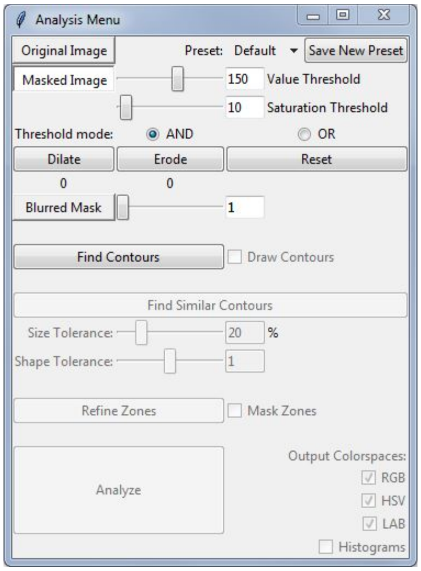

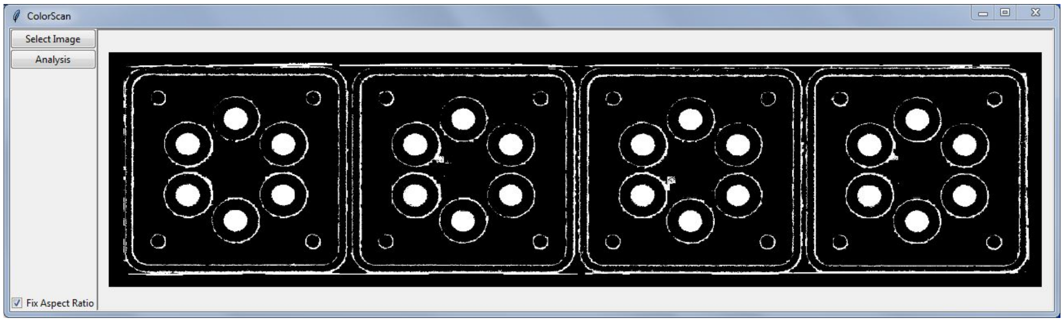

During the masking process, the white areas of the binarized image may be slightly enlarged or reduced using the “Dilate” and “Erode” buttons, respectively. Because this adjustment can change the appearance of the masked image if the buttons are overused, a “Reset” button is included for convenience.

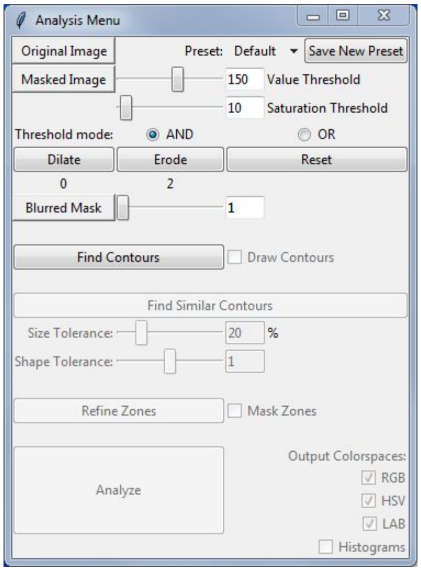

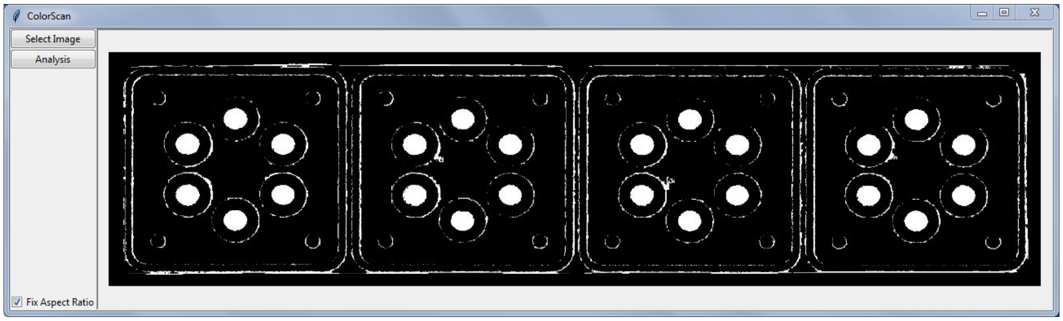

The “Dilate” button can be used to enlarge white areas of the mask that correspond to device output zones, while the “Erode” button can be used to diminish or remove features that were included in the mask but do not need to be analyzed. In the above images, the “Erode” button was used to reduce the size of the white rings that surround the wax barriers of the output zones in the masked image. These undesired features do not need to be removed completely, because

only selected zones are analyzed after contour detection. After this step, the mask is ready for blurring, which reduces the jaggedness of the masked zone edges before contour detection.

Note: The “Threshold mode” radio buttons can be used to adjust the masking protocol. The Value Threshold and Saturation Threshold options each create a mask that excludes pixels below the set threshold. The AND mode requires a pixel to be above both thresholds to be included during the masking step, while the OR mode allows pixels above only one threshold, and both thresholds, to be included. The OR option may be useful for images that do not mask well using the AND option, such as images that contain both very dark, saturated zones and very pale, light zones.

When the “Blurred Mask” button is pressed, the displayed image will update as the blur value is adjusted. Like the Value and Saturation Thresholds, the blur value can be adjusted using its slider, the arrow keys, or by direct text entry. Sufficient blurring should smooth the edges of each masked output zone area so that the contour detection step can correctly recognize the design geometry of the paper-based device.

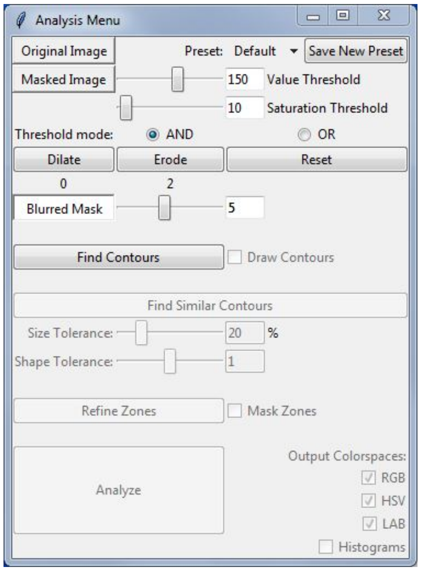

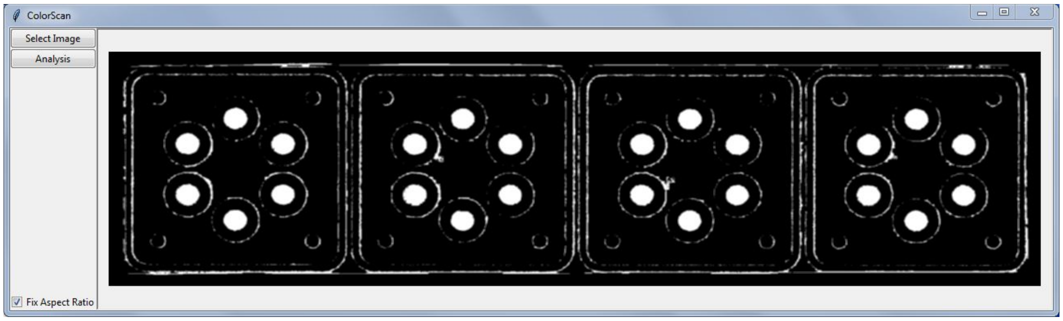

Within ColorScan, masking settings can be saved as named presets to streamline analysis and ensure consistent treatment across multiple images of replicate devices. Once satisfactory masking settings have been identified, click “Save New Preset” to store them as a preset. A small window will open where the preset name can be changed from “Default” to a user-defined name (e.g., “circles1”). Clicking the “Done” button will save the preset, which can be accessed from the drop-down “Preset” menu.

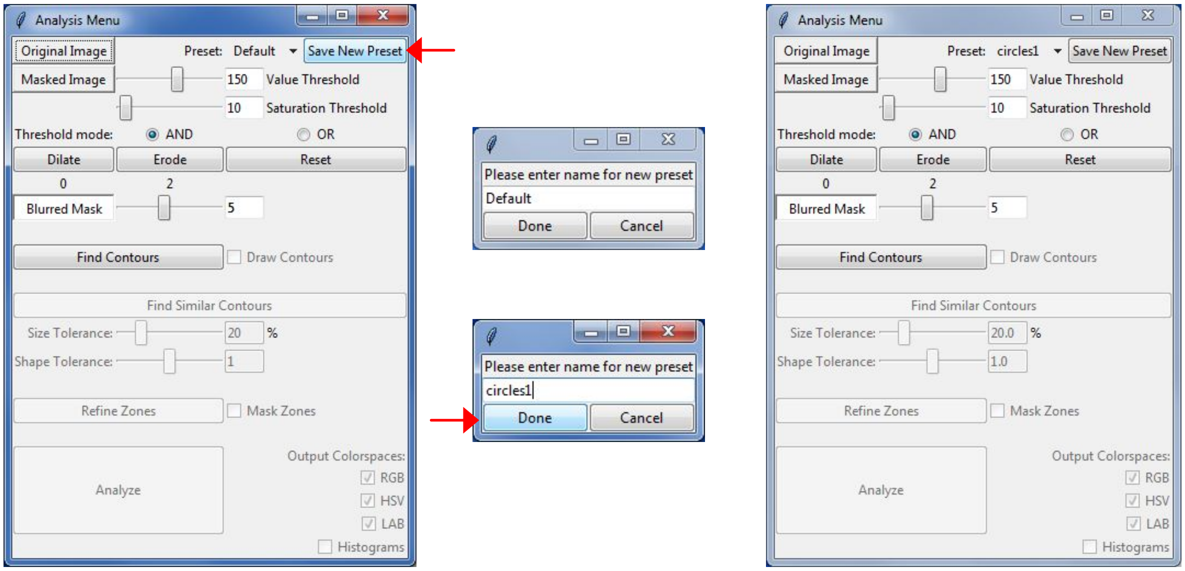

Next, clicking the “Find Contours” button will highlight all of the contours that bound white elements of the masked image. The number of detected contours will be displayed beneath the “Find Contours” button, and the magenta boundary markers may be turned off by unchecking the “Draw Contours” box if masking conditions need to be adjusted.

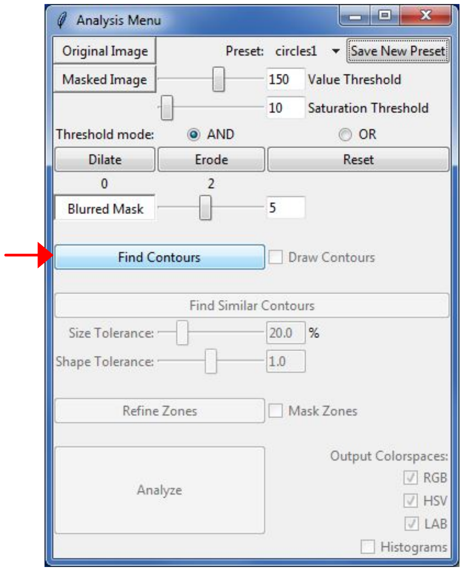

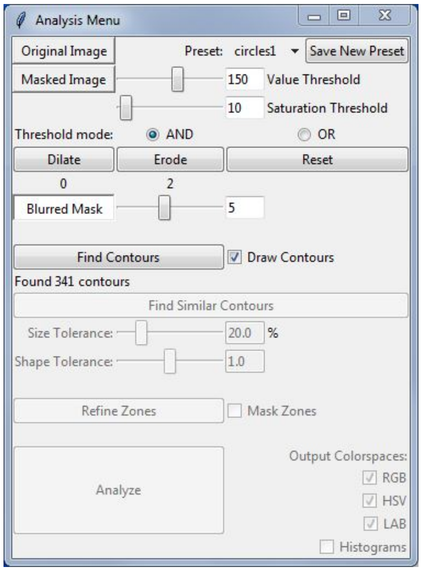

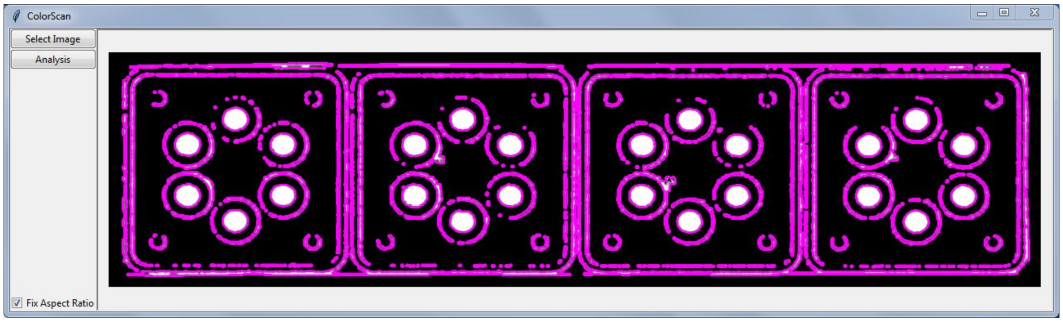

After all of the contours in the image have been identified, contours will turn green when the cursor passes over them. Clicking on a contour that surrounds a device output zone will highlight that contour in yellow. A selected contour can be used as a reference contour by clicking the “Find Similar Contours” button, which becomes available once the reference contour has been selected.

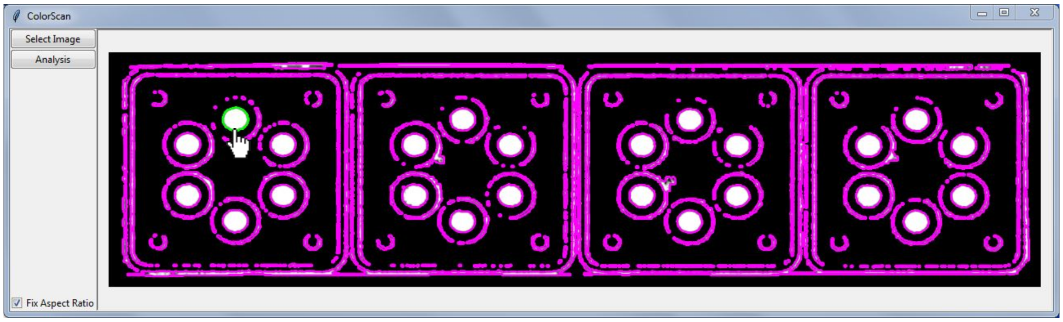

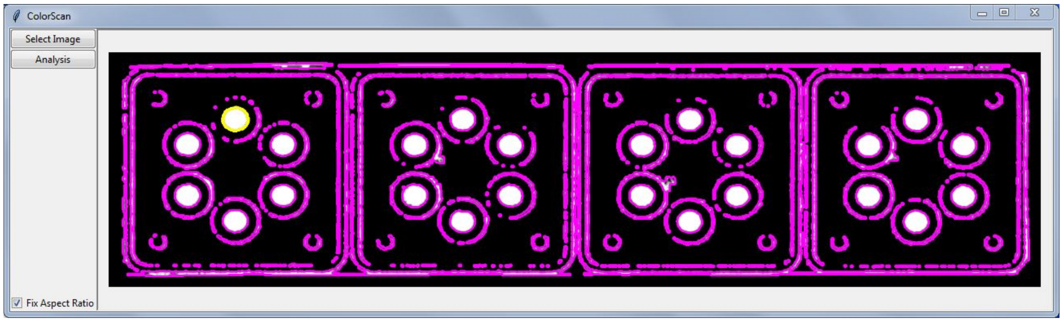

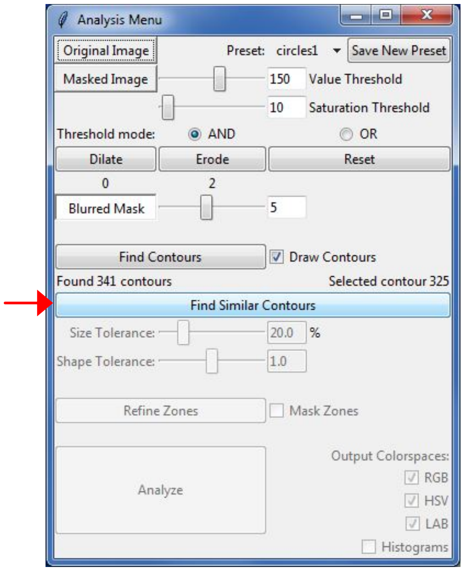

After clicking the “Find Similar Contours” button, contours that meet the Size Tolerance and Shape Tolerance criteria shown beneath the button will be highlighted in light blue. These values can be adjusted using their sliders or text fields, and the included contours will be updated on the masked image as they are adjusted. By default, the Shape Tolerance is set to 20% and the Size Tolerance is set to 1, which should serve as good starting points for including desired contours.

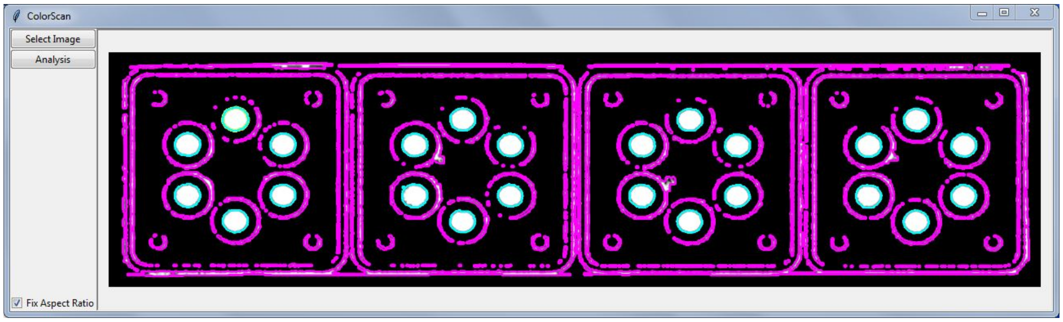

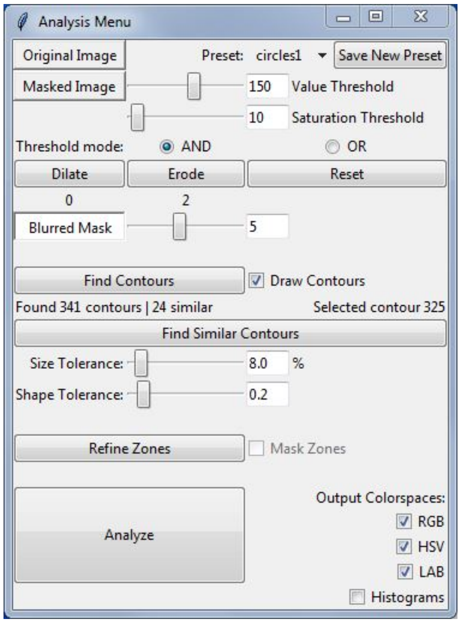

The number of similar contours will be displayed next to the number of total contours and should match the number of output zones to be measured. Once the contours of all of the desired output zones, and only those contours, have been highlighted, the Refine Zones tool will facilitate selection of the exact geometry to be analyzed within each output zone.

If any undesired contours are selected using the “Find Similar Contours” button and are unable to be eliminated with the threshold sliders, they can be deselected by holding the Shift key and clicking on their light blue outlines. This will turn the outlines red, indicating that the areas within these contours will be excluded from the remainder of the analysis process. Similarly, magenta contours that have not been selected for similarity to the reference contour may be added to the analysis set by the same approach. Added contours will turn a dark blue color, indicating that they will be included in the remainder of the analysis process. The total numbers of added and excluded contours will be displayed next to the number of similar contours in the Analysis Menu window with plus and minus signs, respectively.

<u>Note</u>: Named presets can be modified or updated to include additional information at any point. In this example, we can update the preset “circles1” to include the Size Tolerance and Shape Tolerance values used for contour selection.

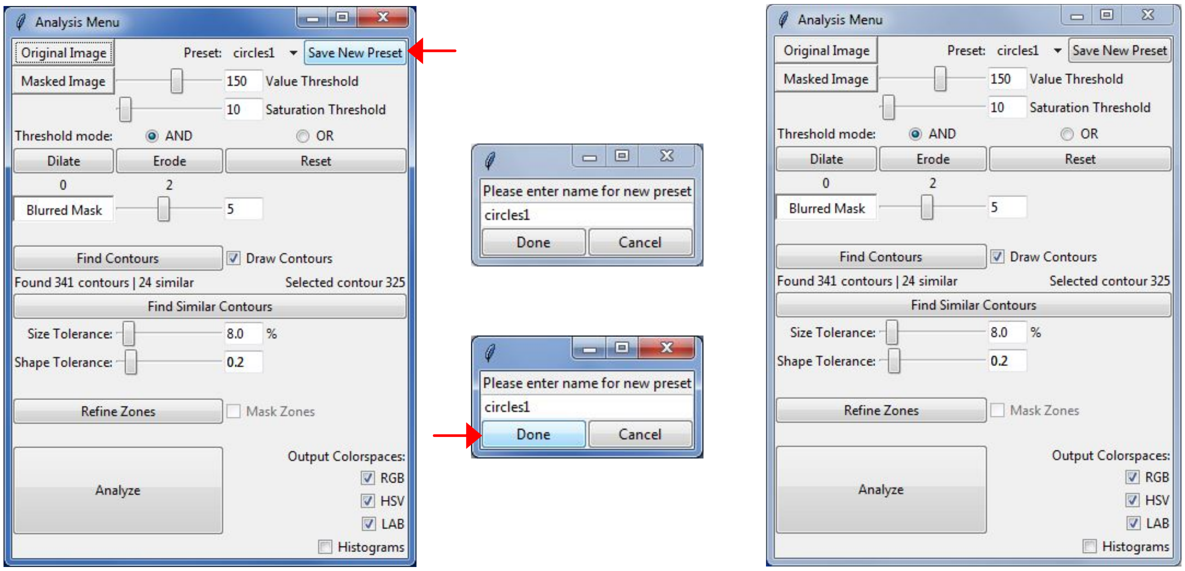

After selecting all of the desired contours for analysis, clicking the “Refine Zones” button will open a window containing an image of the original reference contour and display a modifiable analysis geometry on the device output zone.

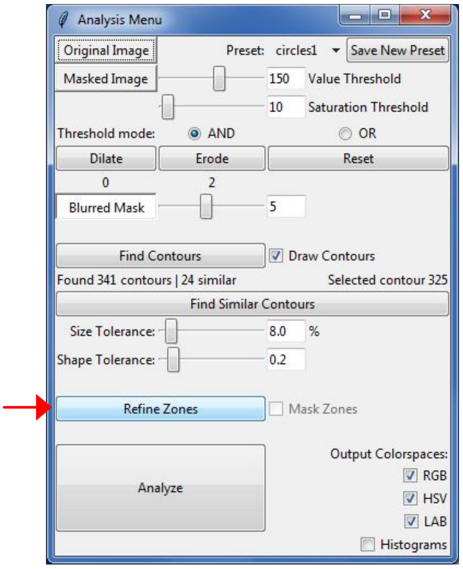

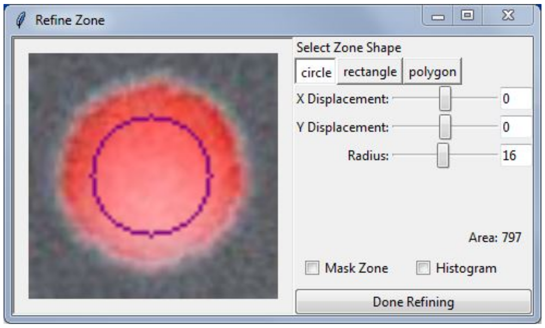

Because our example image has only circular output zones, we are using the “circle” option within the Refine Zones tool. When the Refine Zone window opens, the analysis geometry should be approximately centered on the output zone within the image, but its exact position can be adjusted by using the X Displacement and Y Displacement sliders and fields. Additionally, the Radius slider and field can be used to adjust the exact area of the analysis circle, which is displayed in the Refine Zone window as it is adjusted. This feature allows for the exact analysis area to be easily controlled across multiple analyses of assay replicates or device batches.

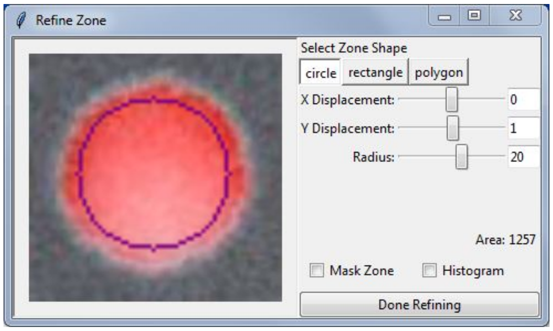

Note: The “rectangle” and “polygon” modes of the Refine Zone window can be used to analyze other zone geometries, which we demonstrate later in this tutorial.

Checking the “Mask Zone” option will change the display to show only the analysis area on a black background. Checking the “Histogram” option will display another window containing a histogram of RGB channel intensities within the analysis area. Within this window, each RGB channel can be turned on or off using a check box, and the intersections between channels can be highlighted using an additional check box. The displayed histogram will automatically update as the analysis zone is scaled or moved within the Refine Zone window, making this tool useful for identifying areas of color localization within the image or determining whether to output histograms during the final analysis step.

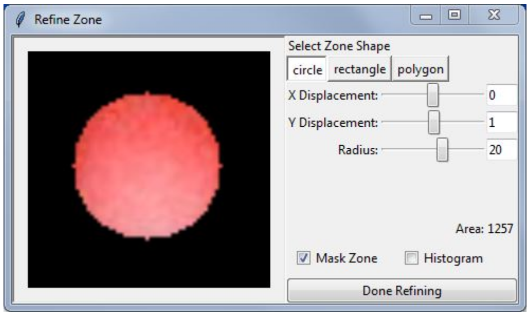

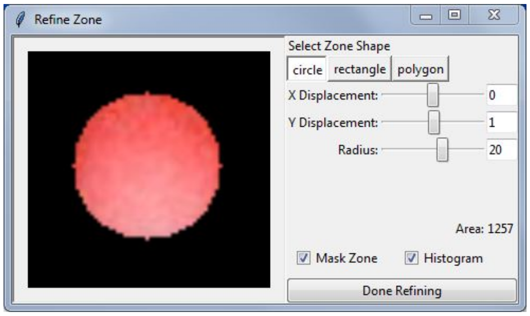

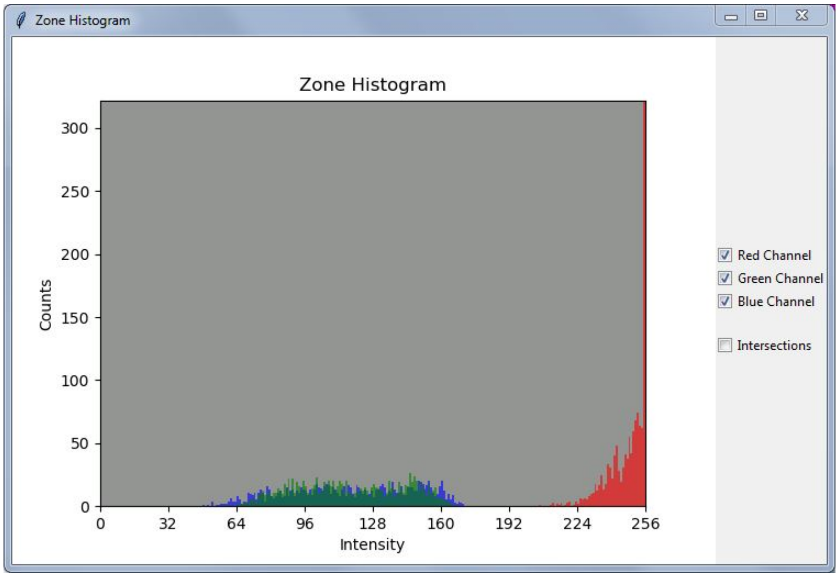

Once the analysis geometry adequately captures the signal contained by the device output zone and does not contain any of the surrounding hydrophobic barrier, click the “Done Refining” button. When the Refine Zone window closes, the device image displayed within the main ColorScan window will show red outlines of the analysis area drawn on each output zone.

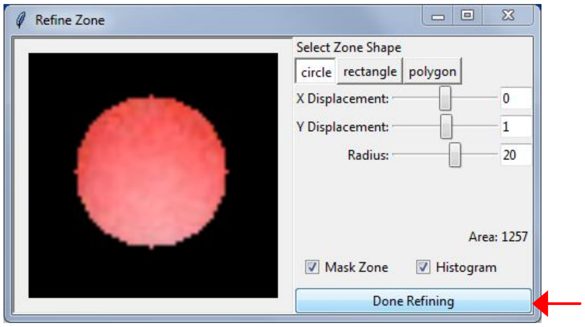

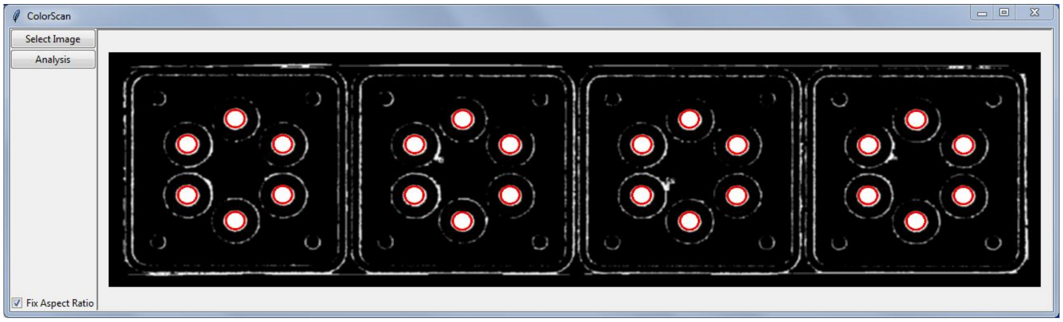

Additionally, checking the “Mask Zones” box within the Analysis Menu window will show all of the regions of the original image that have been selected for measurement. This view can be useful for verifying that each analysis area is satisfactory before completing the analysis.

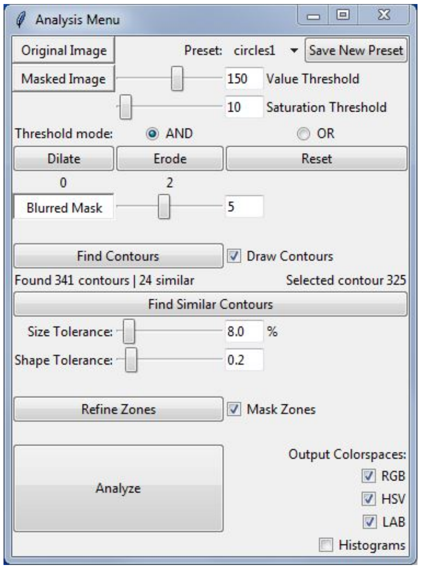

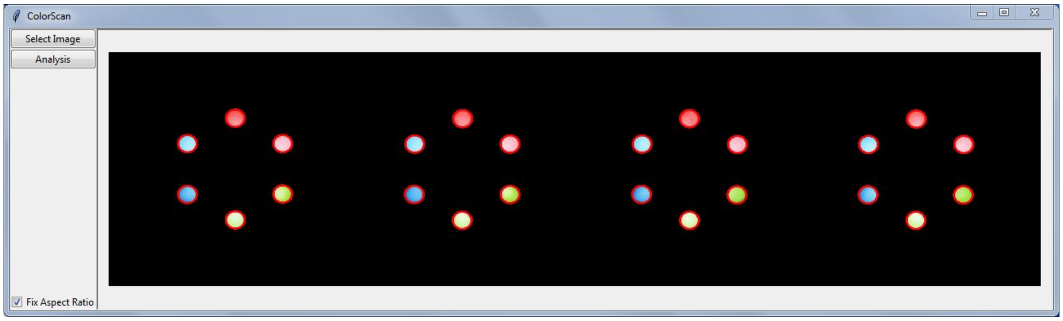

Before clicking the "Analyze" button to automatically complete color intensity measurements for all selected zones, the "Output Colorspaces" check boxes can be used to select the color spaces in which measurements will be performed. The configuration below directs ColorScan to provide mean intensity measurements and their standard deviations in the RGB, HSV, and CIELAB color spaces. After the "Analyze" button is pressed, numbered labels will be displayed next to each analyzed zone. These numbers correspond to the results in the exported .csv file that contains all of the measured values, as well as the exported cropped images of the analyzed zones.

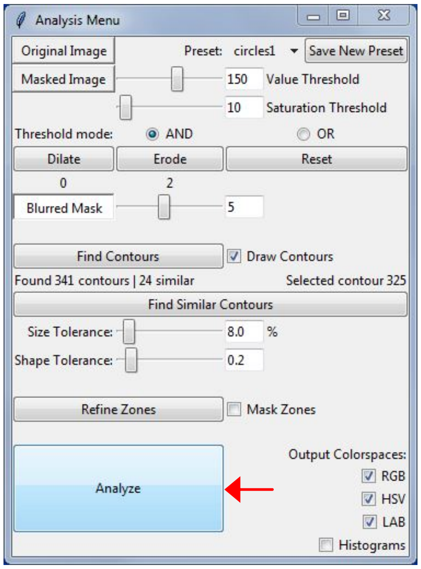

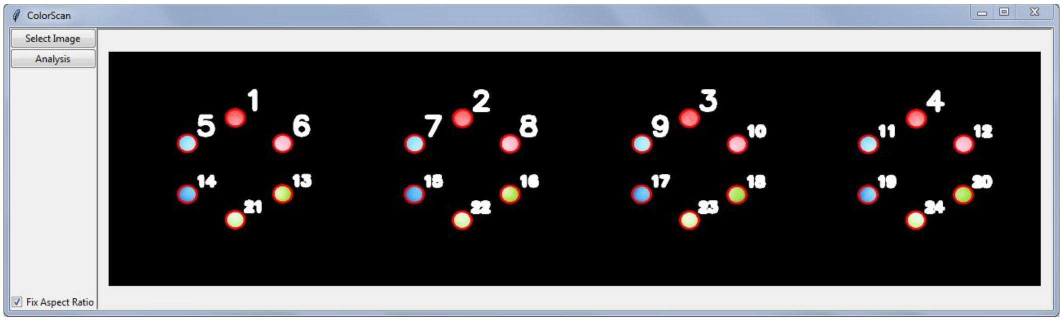

When the total number of analysis zones in the image matches the number of “spots” labeled as “analyzed” within the Python Shell window, analysis is complete. All Python and ColorScan interface windows can then be closed, or additional analyses may be performed on the same image.

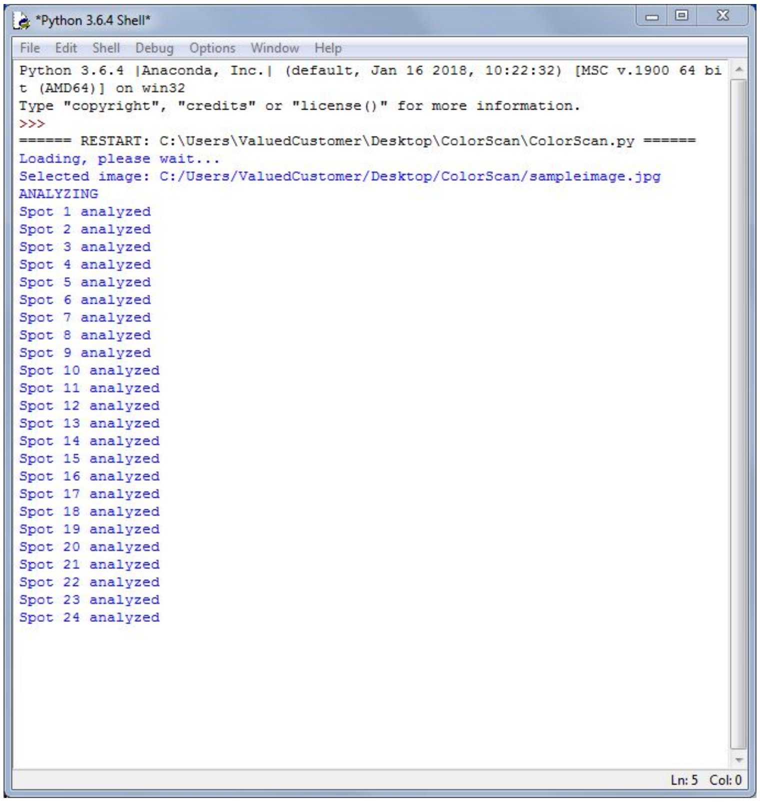

ColorScan Analysis Results

The .csv file of measured values will be exported to a new folder named after the original image with "_analysis" appended (e.g.,"sampleimage_analysis"). This folder will be stored in the same directory as the original image. Subsequent analyses of the same image will produce sequentially numbered output folders (e.g., "sampleimage_analysis_1", "_2", etc.) Additionally, a file called "presets.npy" will appear in the same directory as the "ColorScan.py" file after analysis. This file contains information from any masking and contour finding settings that were saved as presets during the analysis process and must be stored in the same directory as the "ColorScan.py" file for saved presets to work within the interface.

Before analysis:

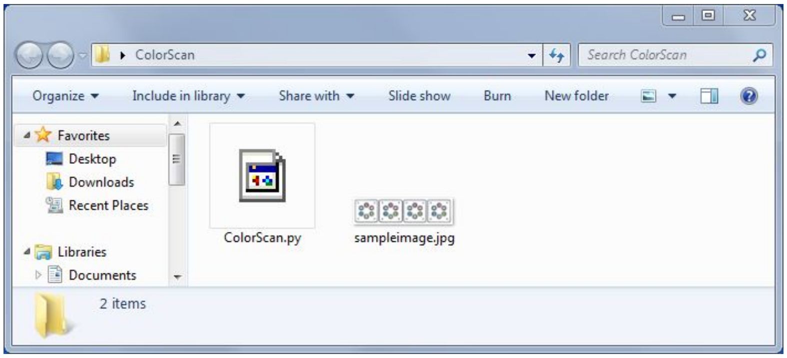

After analysis:

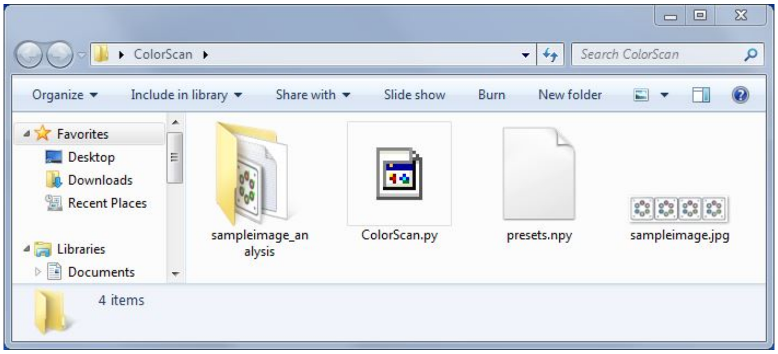

The "_analysis" folder contains four items: a folder called "crops", a "_colors.csv" file containing measured color intensity values (e.g., "sampleimage_colors.csv"), a "_labeled.jpg" version of the original image with numerical labels corresponding to the measured values (e.g., "sampleimage_labeled.jpg"), and a "_mask.jpg" image of the mask that was applied to facilitate contour identification during analysis (e.g., "sampleimage_mask.jpg").

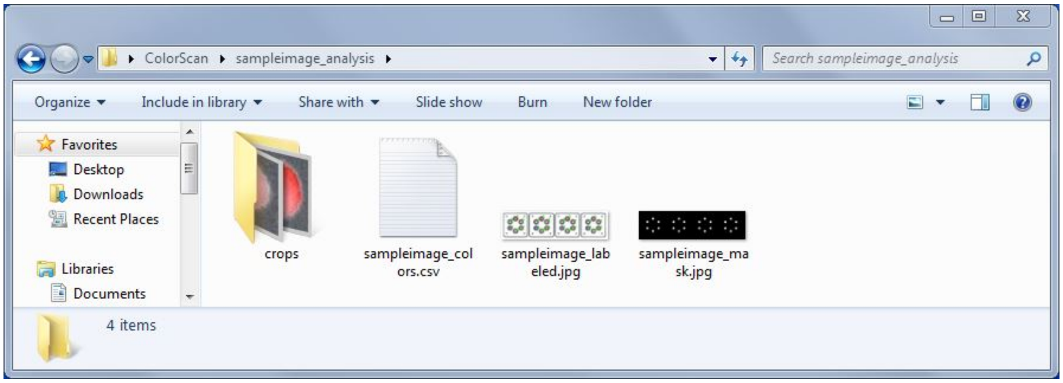

Within the “crops” folder, there is a subfolder called “drawn” and 1 cropped image of each analyzed output zone, named with a number corresponding to the label assigned to it during the analysis process (e.g., “sampleimage_crop_1.jpg”). Each image contains some of the hydrophobic barrier surrounding the output zone to ensure that the entire area of the zone is displayed.

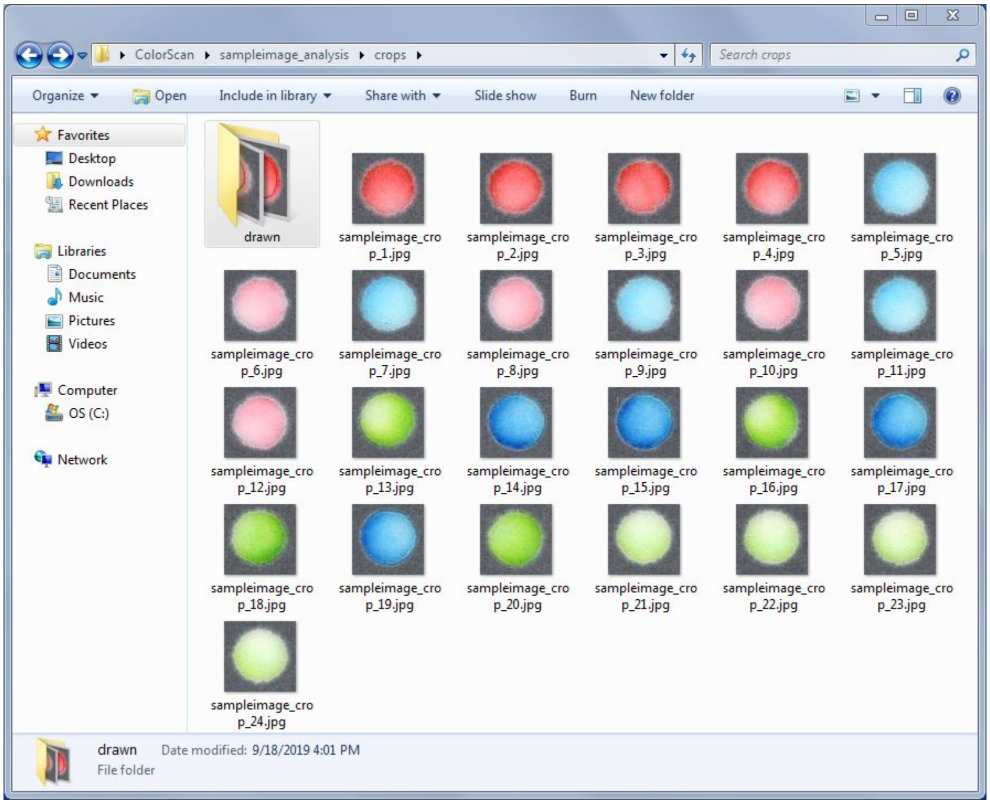

The “drawn” folder contains the same images shown in the “crops” folder, but with the contour of the analysis area drawn on each image in red (e.g., “sampleimage_crop_draw_1.jpg”).

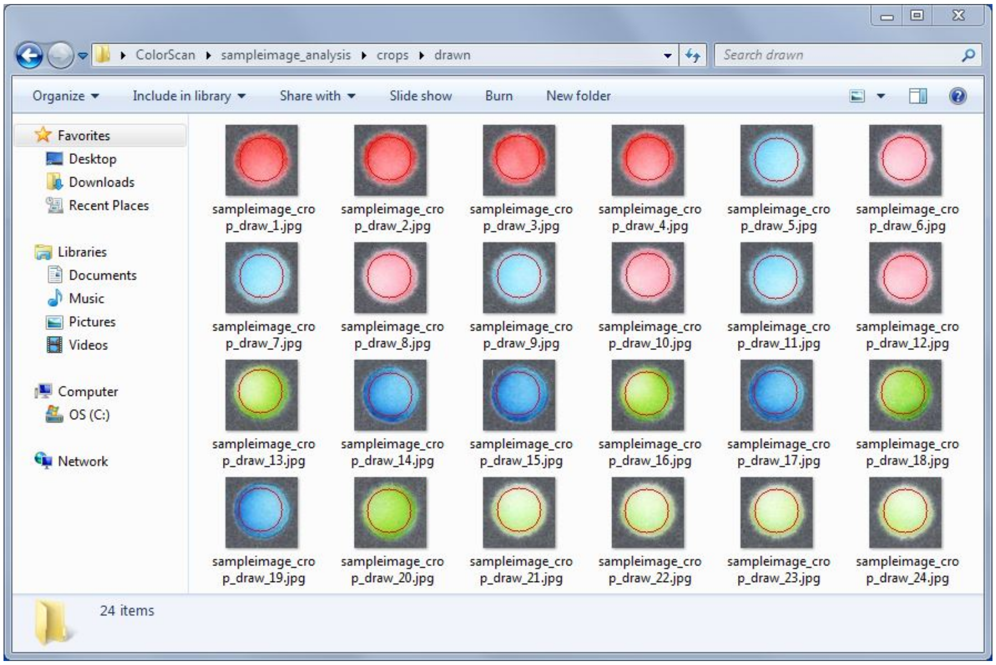

The “_colors.csv” file (e.g., “sampleimage_colors.csv”) is a comma-delimited file that contains the measured color intensity values, organized by the labels assigned in the “_labeled.jpg” (e.g., “samplimage_labeled.jpg”) image. When the file is viewed as a spreadsheet, the numbers in the “id” column correspond to the label assigned to each output zone. For the demonstrated analysis, in which we selected the RGB, HSV, and CIELAB color spaces, the file contains:

(i) RGB and greyscale values for each device output zone

	A	B	C	D	E	F	G
1	id	R	G	B	std R	std G	std B
2	1	246.9331742243	116.5656324582	117.0214797136	8.7158076853	25.5140442914	29.4099548039
3	2	247.1089896579	119.1726332538	121.2012728719	9.4732411489	23.3271421253	26.612127172
4	3	246.84566428	119.1718377088	121.6961018298	8.7664861791	20.4158221485	23.5006374581
5	4	247.1336515513	133.614956245	137.938743039	7.9803630583	28.7338434949	32.2318357475
6	5	156.6101829753	225.2187748608	248.5107398568	22.6935060866	11.1549025618	5.670907558
7	6	249.6229116945	193.4447096261	207.184566428	7.1070494066	14.0020571088	12.695039422
8	7	156.5934765314	224.9793158313	247.8186157518	20.9617082134	10.2346169648	6.4853205756
9	8	249.4645982498	190.0143198091	202.4216388226	6.8495988549	15.5187094782	13.7393201867
10	9	164.9403341289	228.9363564041	248.7740652347	16.1831336485	8.0639305395	6.0360778574
11	10	249.2848050915	187.3396976929	200.0906921241	6.6671065752	14.7567912197	13.1049754954
12	11	160.4836913286	227.0445505171	247.6714399364	22.8429649274	10.3337120509	6.4294287037
13	12	249.9570405728	189.6205250597	202.7820206842	6.1882224595	13.175215711	11.7238265733
14	13	190.0270485282	230.6507557677	119.3221957041	24.7259776358	10.973377844	44.4898825639
15	14	88.3754972156	178.6690533015	234.1980906921	31.062525357	26.306200227	11.4323326686
16	15	78.2816229117	171.3754972156	231.9474940334	26.0073777166	23.56599706	12.0432968117
17	16	182.7557677009	228.3206046142	110.4741447892	26.5475065489	11.5546433217	43.7973510976
18	17	85.307875895	176.3659506762	232.2060461416	29.7863761422	26.172089178	12.0214484151
19	18	164.8607796341	221.3062848051	95.0334128878	24.89364228	12.8109402937	29.5090104851
20	19	97.5592680986	185.3031026253	235.5043754972	35.3032080022	26.3963956626	10.5024233266
21	20	169.9315831344	224.3985680191	94.5298329356	16.6944585185	9.588399474	27.0488984017
22	21	216.338106603	243.3778838504	186.5465393795	11.651233275	7.4910450475	24.5715801622
23	22	216.1487669053	241.969769292	184.6539379475	14.1065154913	8.2176465356	25.2181959885
24	23	218.6523468576	242.0007955449	186.6515513127	13.8119854901	8.6412177243	24.4487250934
25	24	219.1471758154	243.6404136834	189.9809069212	12.8948927768	7.4631668863	26.0748195509

	A	I	J
1	id	Gray	std Gray
2	1	155.5974940334	15.5671148862
3	2	157.6568687351	14.3082502241
4	3	157.6340779634	12.5565268797
5	4	168.0499578361	17.4264982057
6	5	207.3600898966	9.451686988
7	6	211.80833572	8.6119404328
8	7	207.1356300716	8.7132943092
9	8	209.2043874304	9.4673300749
10	9	212.0630445505	6.8039215356
11	10	207.3148981702	9.0133334805
12	11	209.4943190135	9.1641566585
13	12	209.1615536993	8.063638579
14	13	205.8128114558	11.039581325
15	14	158.0015902944	18.0667416527
16	15	150.445636436	15.9283667736
17	16	201.262221957	11.5732240507
18	17	155.5053571997	17.8106563539
19	18	190.0339713604	11.1026402765
20	19	164.7906412092	18.7867147624
21	20	193.3079037391	8.1304189019
22	21	228.8142171838	6.2704500908
23	22	227.7152848051	7.023089888
24	23	228.7097955449	7.1100304671
25	24	230.19975179	6.5493109855

(ii) HSV values for each device output zone

	A	L	M	N	O	P	Q
1	id	H	S	V	std H	std S	std V
2	1	165.6229116945	0.53738905	0.96836539	177.5209675564	0.10586667	0.03417964
3	2	215.0946698488	0.52563683	0.96905486	174.2546480476	0.09084539	0.03714997
4	3	221.7804295943	0.5233469	0.96802221	172.835778597	0.08078666	0.03437838
5	4	261.3301511535	0.4658368	0.96915157	156.4450335361	0.11455879	0.03129554
6	5	194.800318218	0.37013119	0.97455192	2.7110780532	0.08851016	0.02223885
7	6	345.0580747812	0.22492396	0.97891338	3.2859916437	0.05181271	0.02787078
8	7	194.8321400159	0.36843402	0.97183771	2.0935901614	0.08113931	0.02543263
9	8	347.123309467	0.23815184	0.97829254	2.6238437667	0.06086339	0.02686117
10	9	194.1241050119	0.33718315	0.97558457	2.0254230665	0.06116398	0.02367089
11	10	347.492442323	0.24840345	0.97758747	2.4961670222	0.05642699	0.02614552
12	11	193.7470167064	0.35213627	0.97126055	2.6810794844	0.09066648	0.02521345
13	12	346.7764518695	0.24144009	0.98022369	2.4227414624	0.04801833	0.02426754
14	13	81.9029435163	0.48890449	0.90451277	3.1519663533	0.17543555	0.04303285
15	14	202.3929992045	0.6274541	0.91842389	3.6342158132	0.12158906	0.04483268
16	15	203.4383452665	0.6668289	0.90959802	2.8507660289	0.10132753	0.04722861
17	16	83.0739856802	0.52259192	0.89537492	3.3442287327	0.17356268	0.04531233
18	17	202.3452665076	0.63718159	0.91061195	3.7169283492	0.11694718	0.04714293
19	18	86.6507557677	0.57529755	0.86786778	3.4608483909	0.11897244	0.05023898
20	19	201.1455847255	0.59021636	0.92354657	3.5478721754	0.14065147	0.04118597
21	20	85.3285600636	0.58169935	0.87999438	2.2081977687	0.10930962	0.03760157
22	21	89.3508353222	0.23483551	0.95442307	4.6822912905	0.0887978	0.02937665
23	22	87.1614956245	0.2387602	0.94890106	3.2099953764	0.08790974	0.03222606
24	23	85.7342879873	0.2304179	0.94902273	3.9827843716	0.08516866	0.03388713
25	24	88.0493237868	0.22148595	0.9554526	4.7264840518	0.09609215	0.02926732

(iii) CIELAB values for each device output zone

	A	S	T	U	V	W	X
1	id	L	a	b	std L	std a	std b
2	1	65.06621742	49.7581543357	24.5918854415	6.04589323	8.4470590985	8.632773081
3	2	65.57287036	48.9570405728	22.8894192522	5.80743925	6.9410336151	7.3927026134
4	3	65.49643565	48.9260143198	22.4431185362	5.04941082	6.3119716368	6.8047074688
5	4	68.82992497	43.4725536993	18.0389817025	6.97998934	9.7908641481	8.7383558115
6	5	86.06548427	-15.5178997613	-18.0883054893	4.17111808	2.0106809573	5.3281496854
7	6	83.60023086	22.1026252983	0.9832935561	3.91729229	5.094474313	1.7282916928
8	7	85.96596316	-15.6101829753	-17.8408910103	3.85896749	1.7242225043	4.6504016646
9	8	82.67895862	23.1869530628	2.1320604614	4.17717596	6.0536625751	1.7460212671
10	9	87.47905845	-15.2903739061	-16.015115354	3.01989197	2.0755449054	3.3146708444
11	10	81.97794313	24.185361973	2.3548130469	4.00174782	5.6420550274	1.7128261946
12	11	86.73311807	-15.6022275259	-16.6213206046	3.94754693	2.3617061917	5.1085082451
13	12	82.62529833	23.6030230708	1.8361177407	3.66253704	4.7658714876	1.4058181905
14	13	86.80674497	-30.0326173429	48.7398568019	4.9666546	9.3453012834	15.2912309762
15	14	69.6719547	-9.5330151154	-35.7740652347	8.94443095	6.8814764027	8.8690569209
16	15	67.14056187	-8.2163882259	-38.5019888624	8.04045696	6.174287948	7.0109724793
17	16	85.60094841	-32.5632458234	51.2816229117	5.20378011	9.7077101114	14.8070137945
18	17	68.82306144	-9.4908512331	-36.015115354	8.89267024	6.743637491	8.6859240555
19	18	82.33110269	-37.6778042959	54.6372315036	5.21917284	7.0335320701	8.5400724914
20	19	71.97466735	-10.8353221957	-32.9204455052	9.06899362	6.0868097995	9.8027501114
21	20	83.47824731	-37.2593476531	56.2840095465	3.851474	5.1307062778	8.7362852441
22	21	92.91653018	-18.4526650756	24.5441527446	2.95834311	5.7980762159	9.3120481031
23	22	92.48256821	-18.139220366	24.9299920446	3.40384088	5.914441452	8.860262279
24	23	92.70469683	-16.968973747	24.2100238663	3.49184214	5.485048019	8.6728094793
25	24	93.24629136	-17.0739856802	23.2911694511	3.05107814	6.2794964639	10.1416158624

and (iv), the pixel area of each analyzed zone

	A	Z
1	id	Area [pixels]
2	1	1257
3	2	1257
4	3	1257
5	4	1257
6	5	1257
7	6	1257
8	7	1257
9	8	1257
10	9	1257
11	10	1257
12	11	1257
13	12	1257
14	13	1257
15	14	1257
16	15	1257
17	16	1257
18	17	1257
19	18	1257
20	19	1257
21	20	1257
22	21	1257
23	22	1257
24	23	1257
25	24	1257

Histogram Analysis

Device Image: “sampleimage1.jpg” (resolution: 800 dpi)

In addition to the color intensity measurements shown above, checking the “Histograms” box in the Analysis Menu window will provide histogram data for each analyzed zone. To demonstrate this function, we are using an image of a multilayered device comprising six circular zones containing red, green, and blue dyes. The Analysis Menu settings used to analyze this image are shown below. Details of device fabrication are available in the **Materials and Methods** document.

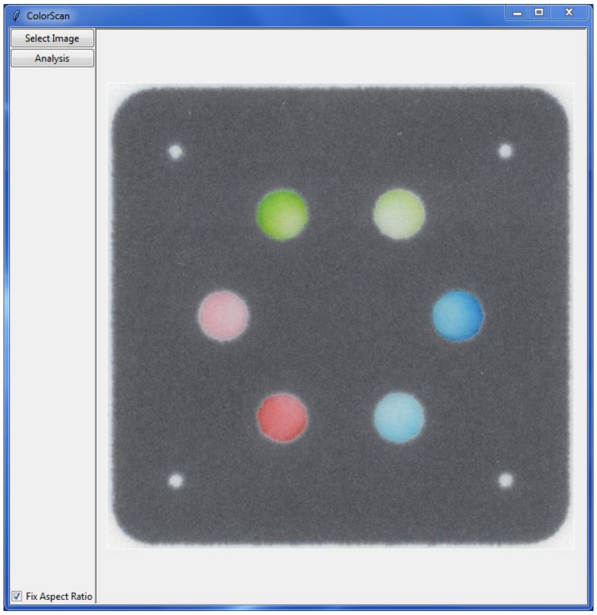

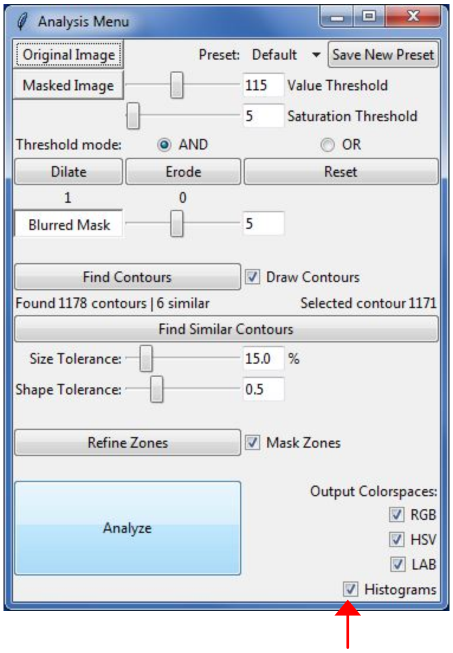

When the Histograms option is selected, an additional folder called “histograms” will be created in the “_analysis” folder.

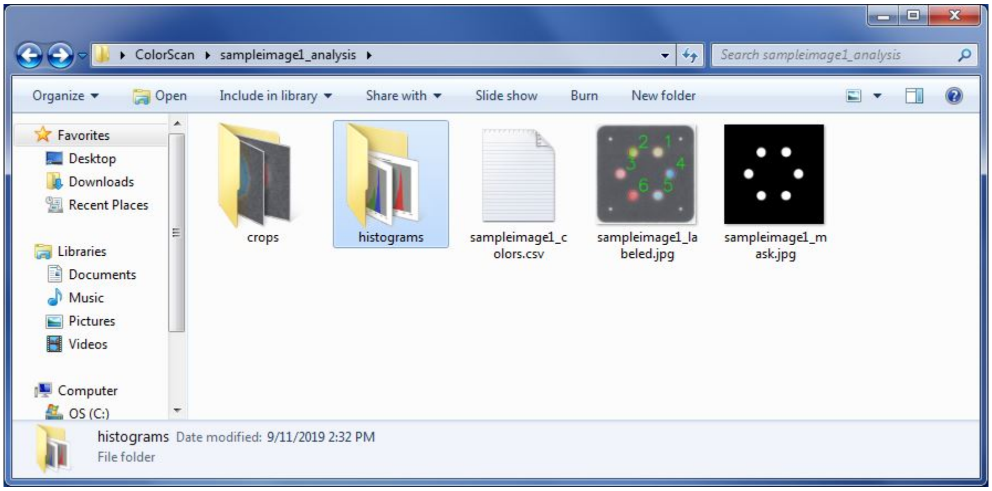

This folder contains .csv files of the histogram data, as well as images of histogram plots labeled as the original image name with “_histogram” and the zone label number appended (e.g., “sampleimage1_histogram_1.csv”).

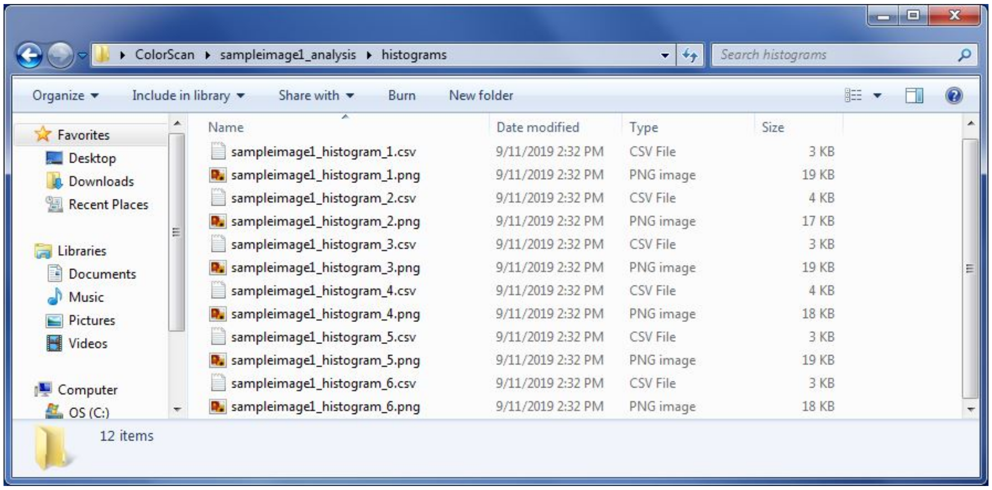

Histogram data are exported as whole number RGB values. When the .csv file is viewed as a spreadsheet, the leftmost column is labeled as “bin”. This column contains all possible RGB intensities, and the Red Channel, Green Channel, and Blue Channel counts for each intensity are organized in the following columns to facilitate further analysis or graphical representation of the data.

	A	B	C	D
1	bin	Red Channel	Green Channel	Blue Channel
112	110	0	0	0
113	111	0	0	0
114	112	0	0	1
115	113	0	0	0
116	114	0	0	1
117	115	0	0	1
118	116	0	0	3
119	117	0	0	1
120	118	0	0	1
121	119	0	0	5
122	120	0	0	8
123	121	0	0	11
124	122	0	0	12
125	123	0	0	21
126	124	0	0	23
127	125	0	0	40
128	126	0	0	29
129	127	0	0	29
130	128	0	0	43
131	129	0	0	51
132	130	0	0	39

Histogram images, plotted in the same format as the histograms displayed using the Refine Zones tool, are provided as .jpg images.

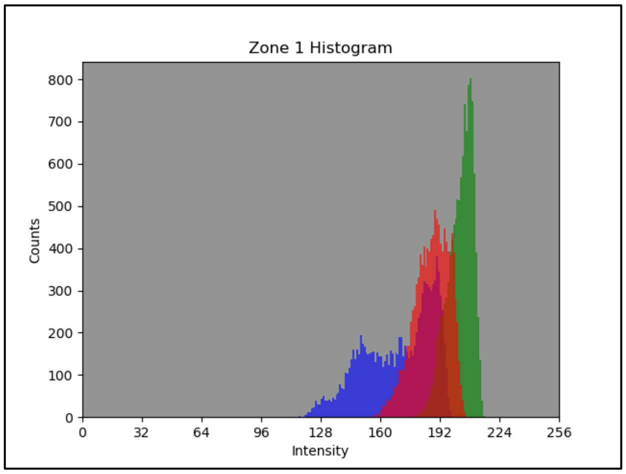

Modes of the “Refine Zones” Tool

Device Images: “refinezones.jpg” (resolution: 800 dpi)

“lateralflowstrip.jpg” (resolution: 800 dpi)

As demonstrated previously, the X Displacement, Y Displacement, and Radius can be adjusted within the “Refine Zone” window for analysis of circular output zones.

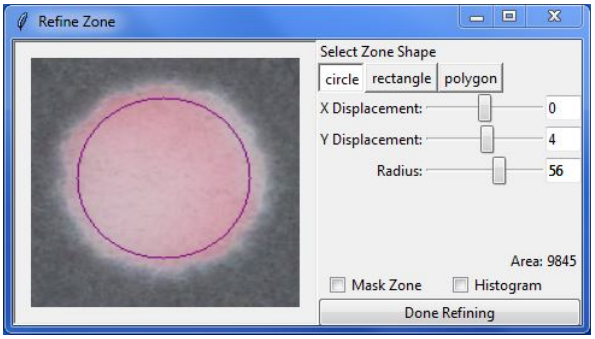

Non-circular zone geometries can be analyzed from the Refine Zone window using the “rectangle” and “polygon” modes. The “rectangle” mode provides familiar X Displacement and Y Displacement options, but instead of a Radius option, it allows the Width and Height of the analysis rectangle to be adjusted. This tool was designed for analysis of lateral flow strips and is useful for images in which a signal-containing line is arranged parallel the sides or top and bottom of the image. In this mode, the analysis rectangle cannot be rotated. Example images of a lateral flow strip signal, scaled for visualization, and how it can be selected using the “rectangle” mode of the Refine Zone window are show below.

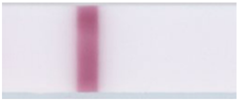

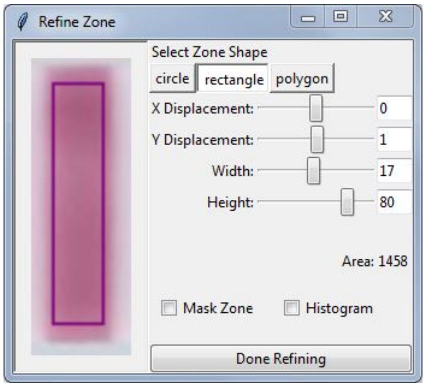

The “polygon” mode allows the user to create a regular polygon to match the geometry of the output zone contained within the reference contour. When this mode is selected, the # Sides slider or field is used to define the shape of the polygon. We limited range of this feature to 6, as symmetrical shapes with more than 6 sides begin to lose resolution and appear rounded when patterned in paper by wax printing and secondary melting. In this mode, the Radius value is used to adjust the size of the analysis area and the Angle value can be used to adjust the rotation of its shape to match the rotation of the output zone within the image. Like the “circle” and “rectangle” modes, the “polygon” mode displays the analysis area within the Refine Zone window to facilitate consist measurement across many devices.

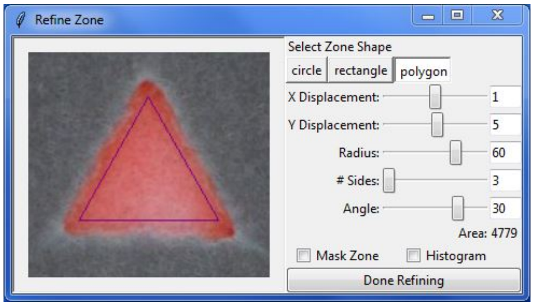

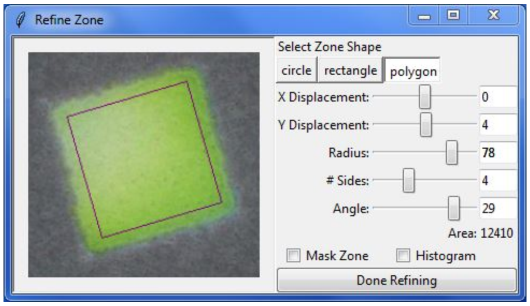

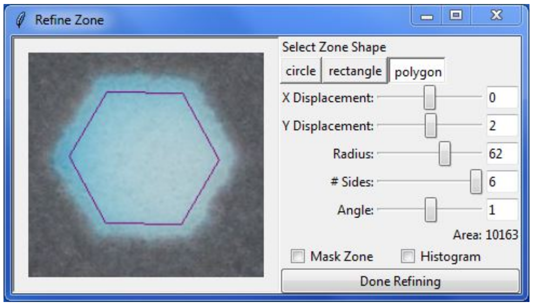

Descriptions of the multilayered paper-based device and the commercial lateral flow test strip used to generate the example images shown above are available in the **Materials and Methods** document.

Analyzing Output Zones at the Ends of Paper Channels

Device Images: “channelends.jpg” (resolution: 800 dpi)

In some paper-based devices, colorimetric indicators are stored at the end of a paper channel. These channels may be used to distribute sample from a proximal sample addition zone or complete a mixing step before a solution is delivered to the device output zones. In ColorScan, the Refine Zones tool can be used to select and analyze the colored regions at the ends of these channels.

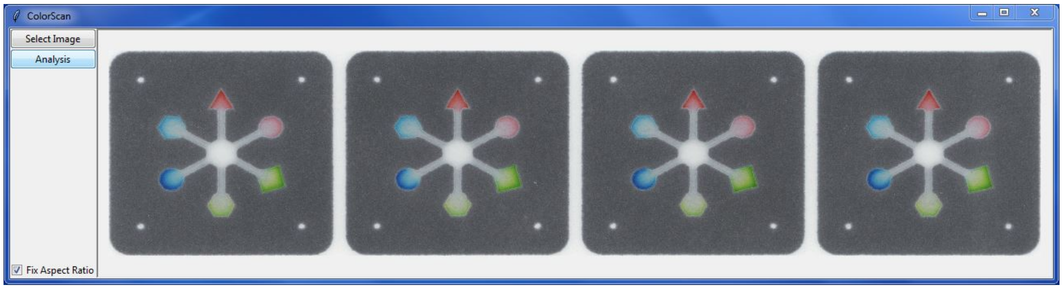

In this example, the Value Threshold can be used to mask the wax-patterned areas of the devices.

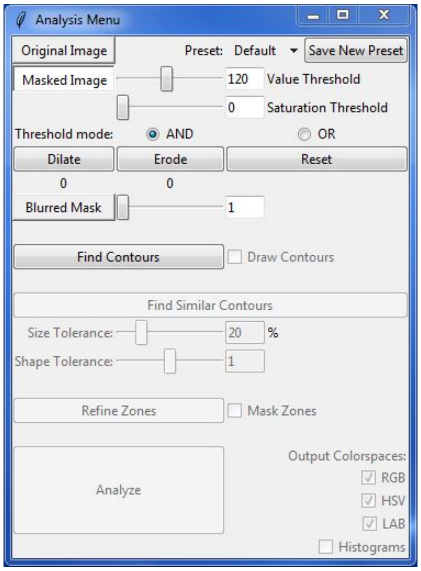

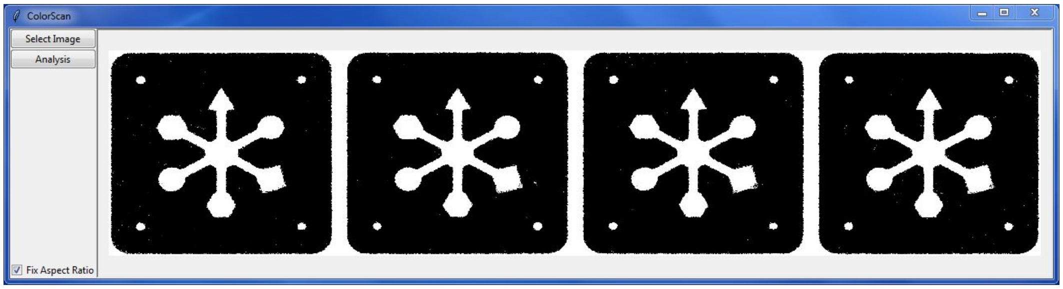

Next, the Saturation Threshold can be used to mask the white areas of the device geometry that do not contain color. For this image, the “Erode” button was also used to minimize granular white features after thresholding. In the resulting masked image, some low color intensity portions of the output zones are excluded from the white regions that will be used for contour detection. These areas can be selected for analysis later using the Refine Zones tool.

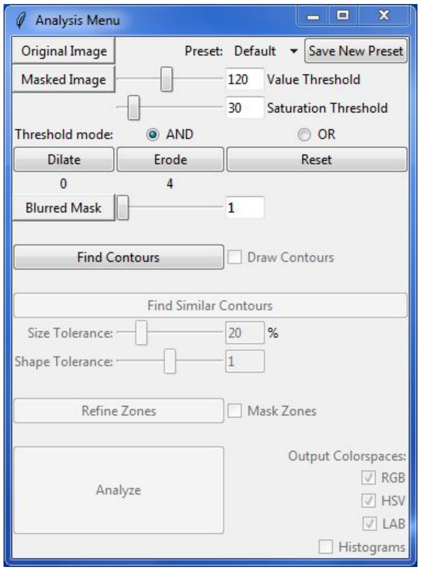

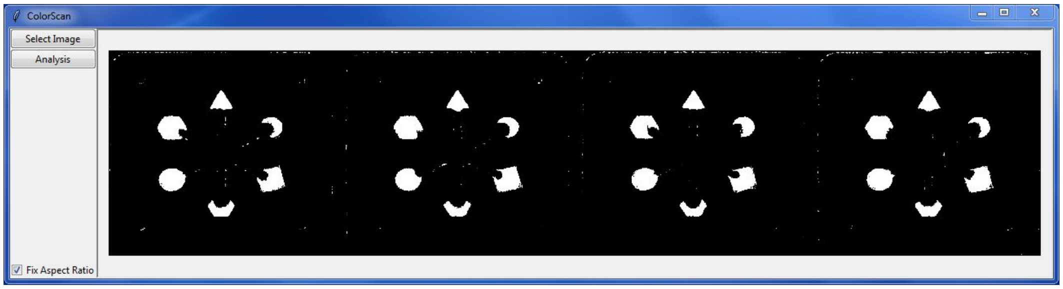

After blurring, the "Find Contours" button identifies closed contours at the ends of each of the radially distributed channels in the image.

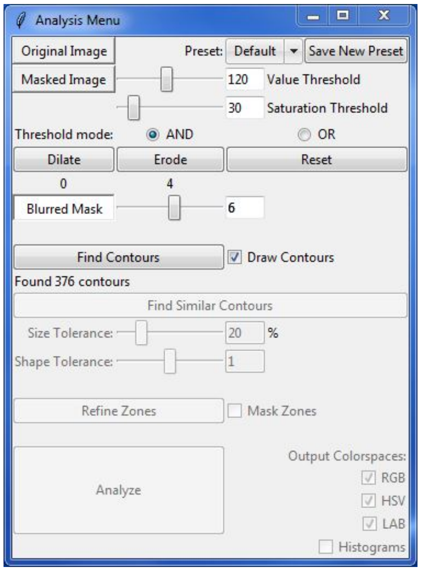

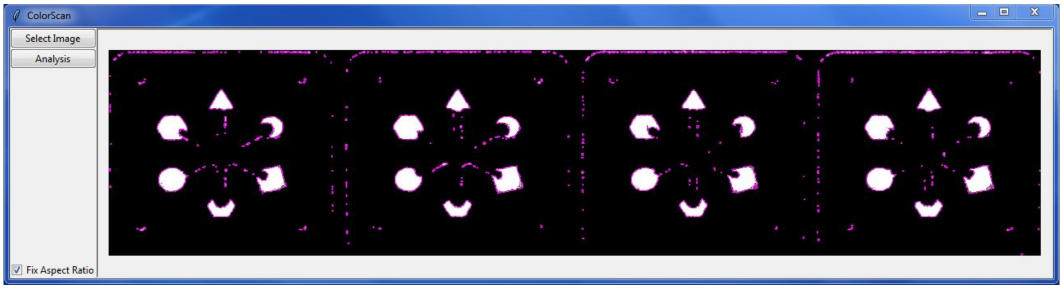

For this demonstration, we will select all of the square zones in the original image for analysis. Clicking on a desired reference contour highlights it in yellow.

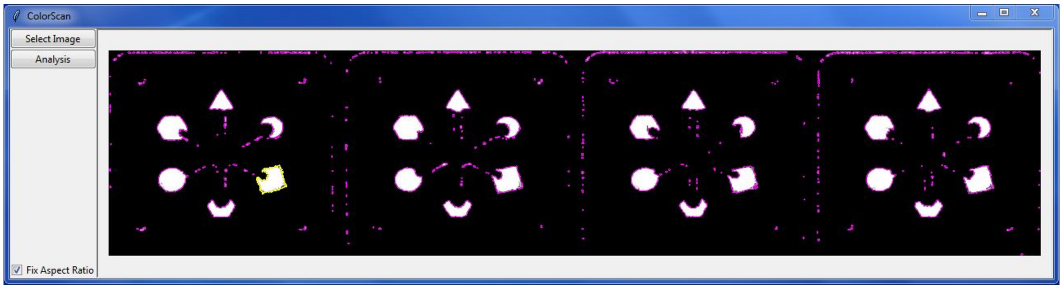

Next, the "Find Similar Contours" button can be used with the Size Tolerance and Shape Tolerance values to select all of the square contours of the device for analysis.

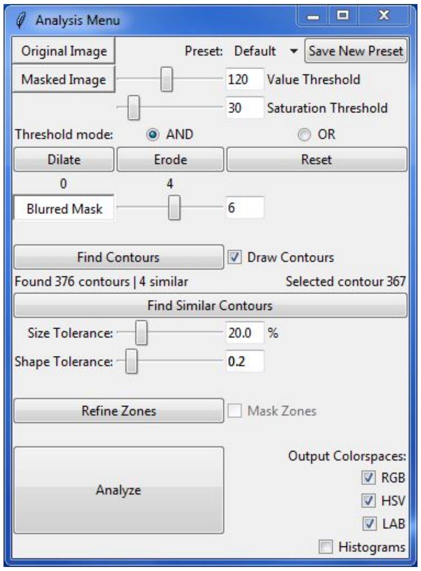

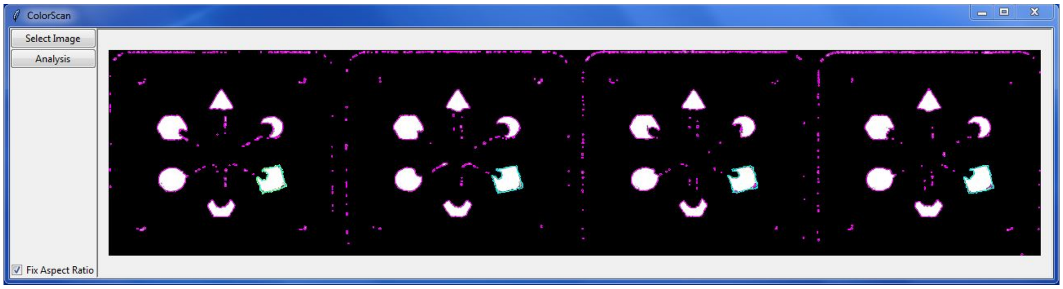

After the desired contours have been selected, the “Refine Zones” tool can be used to specify the size, position, and rotation of the analysis region. Checking the “Mask Zone” box will show only the analysis region.

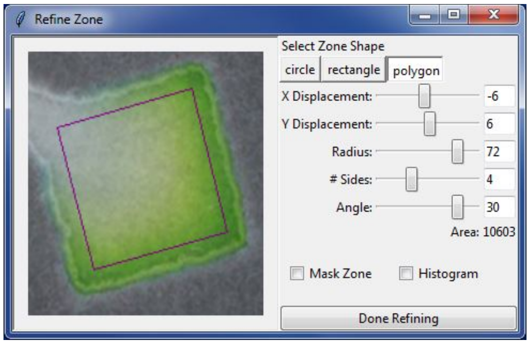

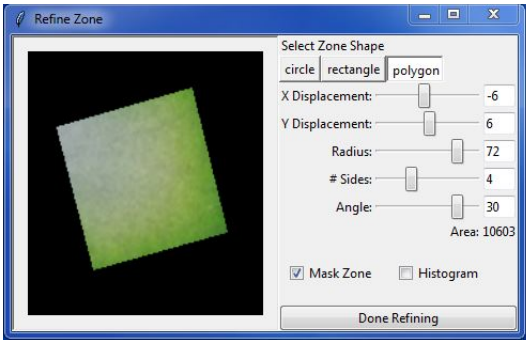

As previously demonstrated, checking the “Histogram” box within the “Refine Zone” window will display a separate window containing a histogram of the analysis region.

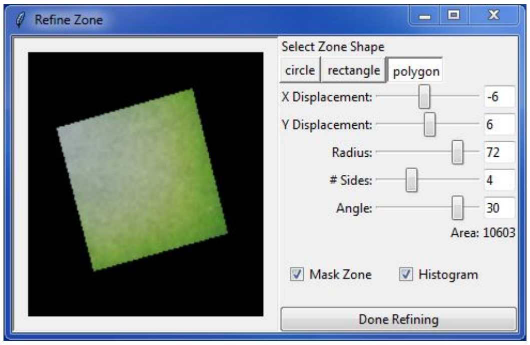

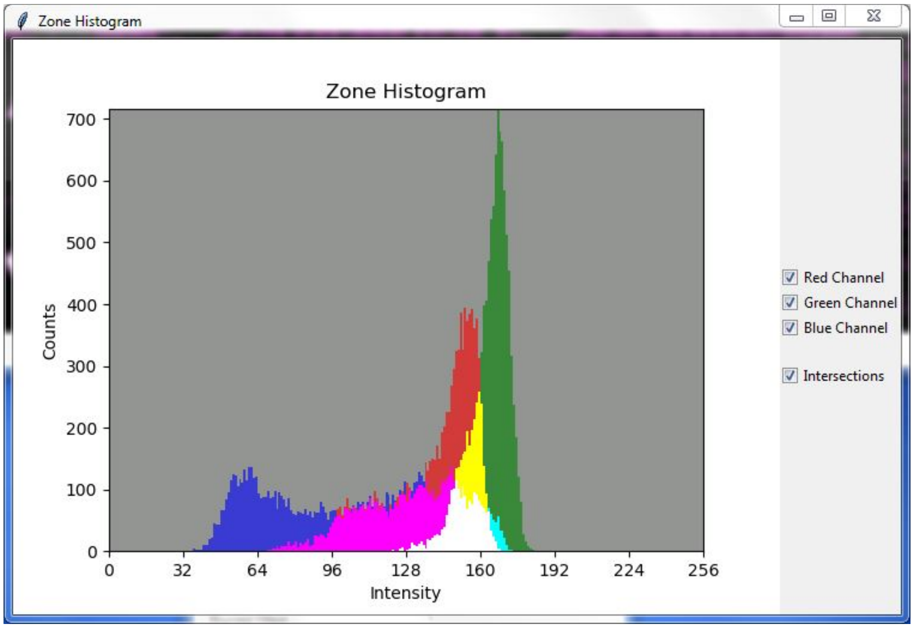

After the “Done Refining” button is pressed within the Refine Zone window, red outlines of the analysis region will be drawn on each selected output zone within the main ColorScan window.

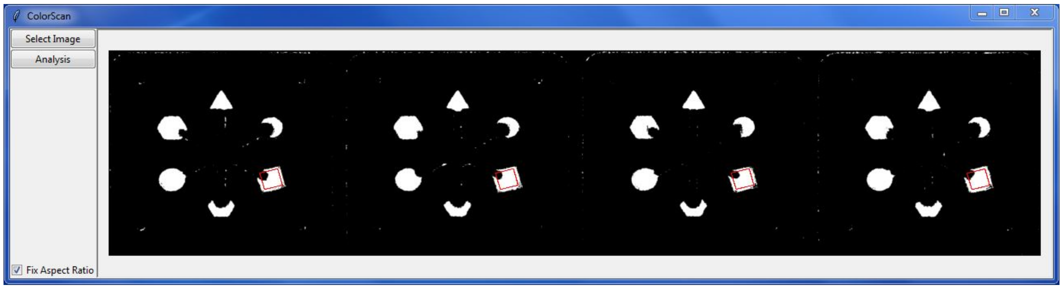

Checking the “Mask Zones” box within the Analysis Menu window will display the regions of the original image that have been selected for analysis.

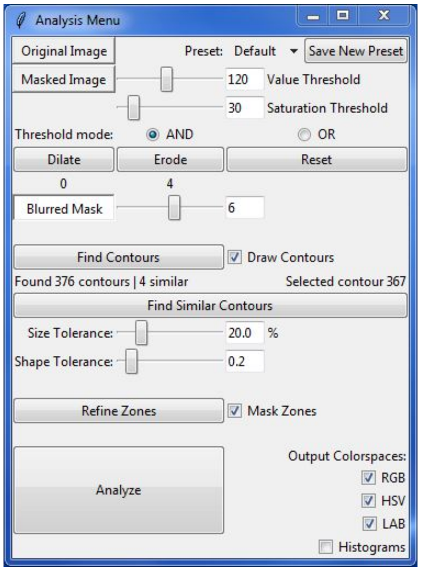

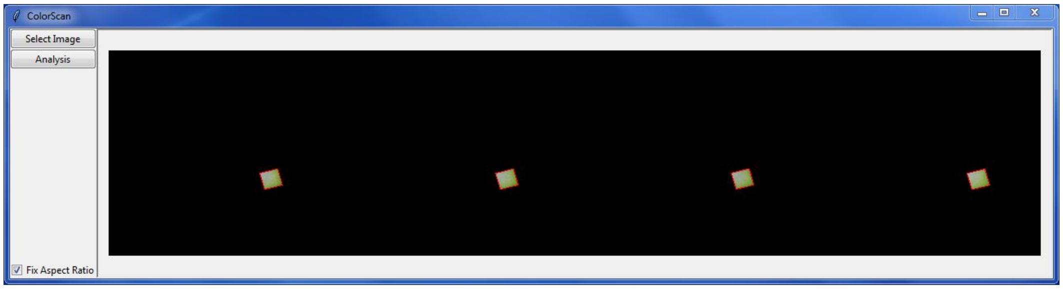

Once the desired analysis regions have been selected, the “Analyze” button can be pressed to complete the measurement process as demonstrated previously.
